# Phenotypic evidence of deltamethrin resistance and identification of selective sweeps in *Anopheles* mosquitoes on the Bijagós Archipelago, Guinea-Bissau

**DOI:** 10.1038/s41598-024-73996-3

**Published:** 2024-10-01

**Authors:** Sophie Moss, Robert T. Jones, Elizabeth Pretorius, Eunice Teixeira da Silva, Matthew Higgins, Mojca Kristan, Holly Acford-Palmer, Emma L. Collins, Amabelia Rodrigues, Sanjeev Krishna, Taane G. Clark, Anna Last, Susana Campino

**Affiliations:** 1 Faculty of Infectious and Tropical Diseases, London School of Hygiene & Tropical Medicine, London, UK; 2 Projecto de Saúde Bandim, Bissau, Guinea-Bissau; 3 Ministério de Saúde Pública, Bissau, Guinea-Bissau; 4 Clinical Academic Group, Institute for Infection and Immunity, and St, George’s University Hospitals NHS Foundation Trust, St. George’s University of London, London, UK; 5 Centre de Recherches Médicales de Lambaréné (CERMEL), Lambaréné, Gabon; 6 Institut für Tropenmedizin Universitätsklinikum Tübingen, Tübingen, Germany; 7 Faculty of Epidemiology and Population Health, London School of Hygiene & Tropical Medicine, London, UK

**Keywords:** Malaria, Evolutionary biology

## Abstract

Vector control in the Bijagós Archipelago of Guinea-Bissau currently relies on pyrethroid insecticide-treated nets. However, data on insecticide resistance in Guinea-Bissau is limited. This study identified deltamethrin resistance in the *Anopheles gambiae* sensu lato complex on Bubaque island using WHO tube tests in November 2022. Whole genome sequencing of *An. gambiae* sensu stricto mosquitoes identified six single nucleotide polymorphisms (SNPs) previously associated with, or putatively associated with, insecticide resistance: T791M, L995F, N1570Y, A1746S and P1874L in the *vgsc* gene, and L119V in the *gste2* gene. Twenty additional non-synonymous SNPs were identified in insecticide-resistance associated genes. Four of these SNPs were present at frequencies over 5% in the population: T154S, I126F and G26S in the *vgsc* gene and A65S in *ace1.* Genome wide selection scans using Garud’s H_12_ statistic identified two selective sweeps: one in chromosome X and one in chromosome 2R. Both selective sweeps overlap with metabolic genes previously associated with insecticide resistance, including *cyp9k1* and the *cyp6aa/cyp6p* gene cluster. This study presents the first phenotypic testing for deltamethrin resistance and the first whole genome sequence data for *Anophelesgambiae* mosquitoes from the Bijagós, contributing data of significance for vector control policy in this region.

**Supplementary Information:**

The online version contains supplementary material available at 10.1038/s41598-024-73996-3.

## Introduction

Insecticide Treated Nets (ITNs) are the most effective method of reducing malaria cases to date, accounting for an estimated 68% of the reduction in malaria cases between 2000 and 2015^1^. Currently, all ITNs contain pyrethroid insecticides^2^. However, the evolution of resistance to pyrethroids in *Anopheles* mosquitoes threatens the efficacy of these ITNs. The World Health Organization (WHO) recommends that countries monitor insecticide resistance in disease vectors as part of the development of insecticide resistance monitoring and management plans (IRMMPs)^3^, assisting national control programmes to make evidence-based decisions about which insecticides to use. Of the 88 countries that reported insecticide resistance data to WHO between 2010 and 2020, 87% reported resistance to pyrethroids in at least one malaria vector, and 33% of countries reported resistance to all four insecticide classes commonly used in vector control^4^. The gold standard for monitoring insecticide resistance is phenotypic testing using WHO tube tests or bottle bioassays^5^, which are time and resource intensive. However, the identification and monitoring of molecular markers associated with resistance could supplement these phenotypic bioassays. Furthermore, the rapid development of sequencing technologies makes whole genome sequencing (WGS) a promising approach for identifying and monitoring the spread of molecular markers on a large scale. Previous studies using WGS data have identified regions of the *Anopheles gambiae* genome associated with pyrethroid insecticide resistance. These include studies conducted by the *Anopheles gambiae* 1000 Genomes Consortium (Ag1000G)^6,7^. Key regions of the genome that have previously been associated with pyrethroid resistance include the cytochrome P450 genes, with the *cyp6aa1* and *cyp9k1* genes demonstrating the strongest signals of association with deltamethrin resistance^6,8^. In addition, the *Tep* family of immune related genes has been found to be associated with deltamethrin resistance^6^.

The most well understood mechanisms of insecticide resistance are metabolic and target-site resistance. Metabolic resistance occurs when mosquitoes evolve the ability to rapidly detoxify insecticides, which is attributed primarily to three different enzyme families; the cytochrome P450s, esterases and glutathione-S-transferases (GSTs)^9^. Whereas, target-site resistance occurs through modifications to the binding sites of insect protein receptors, resulting in conformational changes which impede insecticide binding. Other mechanisms that are less well studied include behavioural resistance, such as mosquitoes evolving to bite during the day when people are not protected by ITNs, and cuticular resistance, which is the evolution of a thickened or altered cuticle that is more difficult for insecticides to penetrate^5^. Pyrethroid resistance in *Anopheles gambiae* is associated with target-site mutations in the voltage-gated sodium channel (*vgsc*) gene, also known as knock-down or “kdr” mutations. These mutations include the L995F^10^ (kdr-west) allele (position 1014 in *Musca domestica*), which is widespread in West and Central Africa, and the L995S^11^ (kdr-east) allele, which is widespread in East Africa^4^. Another well characterised *vgsc* mutation, N1570Y, has been associated with increased levels of pyrethroid resistance when associated with L995F^12^. In addition, L119V in the glutathione S-transferase epsilon class 2 (*gste2)* gene, has been associated with increased resistance to the pyrethroid permethrin^13^. Metabolically mediated pyrethroid resistance has been associated with copy number variations (CNVs) in multiple genes involved in the metabolism of insecticides. This includes CNVs in the cytochrome P450 genes *cyp6aa1*^14^, *cyp6p3*, *cyp6m2*, *cyp6z1*, *cyp9k1*^15,16^, and the *gste2* gene, all of which have been shown to metabolise insecticides^14,17^.

Malaria is a persistent public health problem on the Bijagós Archipelago of Guinea-Bissau, where the peak prevalence of *Plasmodium falciparum* parasitaemia is up to 15%^18^. ITNs are the only vector control intervention currently used on the islands. Of these ITNs, 90% are PermaNet^®^ 2.0, which are impregnated with the pyrethroid insecticide deltamethrin^19^. However, there is little data available about insecticide resistance on the Bijagós or on mainland Guinea-Bissau^20^. A survey on Bubaque island in 2017 identified *An. gambiae* s.s. as the primary malaria vector during the rainy season in June and July, and a majority of *An. melas* during November and December^21^. The Bijagós is also a site of extremely high hybridisation rates between *An. gambiae* s.s. and *An. coluzzii.* These hybrids have been consistently recorded at proportions over 20% since 1995 ^22,23^, which is much higher than found elsewhere in West and Central Africa, where rates of hybridisation are usually below 1%^24^. A recent study suggests that these hybrid mosquitoes may be a novel, cryptic taxon provisionally named the “Bissau molecular form”^25^. Previously, we identified a low prevalence of molecular markers associated with insecticide resistance on the Archipelago, using multiplex amplicon sequencing of *An. gambiae* sensu lato (s.l.) mosquitoes collected in 2019 from 13 different islands. Of 17 mutations screened, we identified three *vgsc* mutations previously or putatively associated with pyrethroid resistance: L995F, N1570Y, A1746S, and the *rdl* mutation A269G which is associated with resistance to the organochlorine dieldrin^19^. The present study builds on this research by: (1) using WHO tube tests to conduct insecticide resistance bioassays for deltamethrin resistance, which have not been conducted on the Bijagós islands previously, and (2) generating whole-genome sequence data for deltamethrin resistant and susceptible individuals of the major vector *Anopheles gambiae* s.s., to identify molecular markers in insecticide resistance genes. This study contributes the first whole genome sequencing data of *Anophelesgambiae* s.s. vectors from the Bijagós.

## Results

### Phenotypic insecticide resistance assay results

Phenotypic tests were conducted in November 2022, investigating the status of resistance to discriminating concentrations and 5-times (5x) intensity concentrations of deltamethrin in *Anopheles gambiae* s.l. mosquitoes. WHO tube tests were conducted according to WHO guidelines^5^. Combined assay results are provided in Table 1. Mosquito mortality in control tests 24 h post-exposure was ≤ 20% for each test conducted. Corrected treatment mortality (%) was calculated using Abbott’s formula when control mortality was ≥ 5%, in line with WHO guidelines^5^.

**Table 1:** Results of WHO tube tests measuring resistance to deltamethrin. Discriminating concentration testing using 0.05% deltamethrin and 5x intensity concentration testing with 0.25% deltamethrin. Tests were conducted in November 2022 during the peak malaria transmission season.

Treatment	24 h post-treatment exposure			
	Alive	Knocked down	Mortality (%)	Corrected treatment mortality (%)
Control (*n* = 66)	61	5	7.58	
Deltamethrin 0.05% (*n* = 101)	51	50	49.50	45.36
Control (*n* = 37)	33	4	10.81	
Deltamethrin 0.25% (*n* = 45)	3	42	93.33	92.52

The total number of mosquitoes exposed to deltamethrin at the discriminating concentration of 0.05% over all replicates was 101, which meets the WHO recommended optimal sample size for this bioassay^5^. Corrected treatment mortality at this concentration (0.05%) was 45.35% (Table 1), which confirmed resistance to deltamethrin^5^. Intensity of resistance was then investigated with 0.25% deltamethrin, which is five times (5x) the discrimination concentration. Corrected treatment mortality at this concentration (0.25%) was 92.52% (Table 1), indicating moderate to high intensity resistance according to WHO guidelines^5^. Due to logistical constraints during fieldwork, the sample size for the intensity resistance testing was *n* = 45, which is lower than the WHO recommended sample size for intensity testing. Therefore, although these tests confirm the presence of deltamethrin resistance, additional tests are required to confirm the resistance intensity^5^.

### Whole genome sequencing of *Anopheles gambiae* s.s. mosquitoes

Whole genome sequencing was conducted with *Anopheles gambiae* s.s mosquitoes for 23 resistant and 10 susceptible mosquitoes (tested with 0.05% deltamethrin during WHO tube tests), and 9 control mosquitoes (exposed to oil instead of insecticide). Variants were called and filtered for quality, resulting in a total of 16,452,859 high quality variants for analysis.

## Allele frequencies of insecticide resistance SNPs

Allele frequencies of 27 candidate SNPs associated with resistance across the *vgsc*, *gste2*, *rdl*, and *ace1* genes were investigated (Supplementary Data 1). Six of these SNPs were identified in the Bijagós whole genome sequence data (Table 2). The *vgsc* T791M and A1746S mutations were identified in susceptible and control mosquitoes at frequencies between 5.0 and 6.3%, but were absent in resistant mosquitoes. The *vgsc* L995F mutation was found at similar frequencies in both resistant, susceptible, and control mosquitoes, at frequencies between 15.0 and 16.9%. The N1570Y mutation was identified at a frequency of 10.9% in resistant mosquitoes and 6.3% in control mosquitoes, with no susceptible mosquitoes carrying this mutation. The P1874L allele was identified in resistant, susceptible and control mosquitoes at frequencies of 4.3%, 10.0% and 6.3% respectively. The *gste2* L119V mutation was identified in resistant mosquitoes at a frequency of 6.8% and was not identified in any susceptible or control mosquitoes. Additional genotype data is provided per mosquito in Supplementary Data 1. Odds ratios with 95% confidence intervals were calculated and no significant associations were found between the resistance phenotype and these SNPs in this sample set, which is likely due to the small sample size (Supplementary Data 3).

**Table 2:** Candidate resistance SNPs identified in *Anopheles gambiae s.s.* mosquitoes. The table includes the alternate allele frequencies and total number of alleles called, arranged by insecticide resistance phenotype.

Chromosome	Gene	Position	SNP	Resistant mosquitoes (*n* = 23)		Susceptible mosquitoes (*n* = 10)		Control mosquitoes (*n* = 9)	
				Alternate allele frequency (%)	Total alleles called	Alternate allele frequency (%)	Total alleles called	Alternate allele frequency (%)	Total alleles called
2 L	*vgsc*	2,416,980	T791M	0	36	5.6	18	5.6	18
2 L	*vgsc*	2,422,652	L995F	15.9	44	15.0	20	16.7	18
2 L	*vgsc*	2,429,745	N1570Y	10.9	46	0	20	6.3	16
2 L	*vgsc*	2,430,424	A1746S	0	46	5.0	20	6.3	16
2 L	*vgsc*	2,430,881	P1874L	4.3	46	10.0	20	6.3	16
3R	*gste2*	28,598,062	L119V	6.8	44	0	18	0	18

### Linkage disequilibrium of *vgsc* insecticide-resistance mutations

**Fig. 1:**
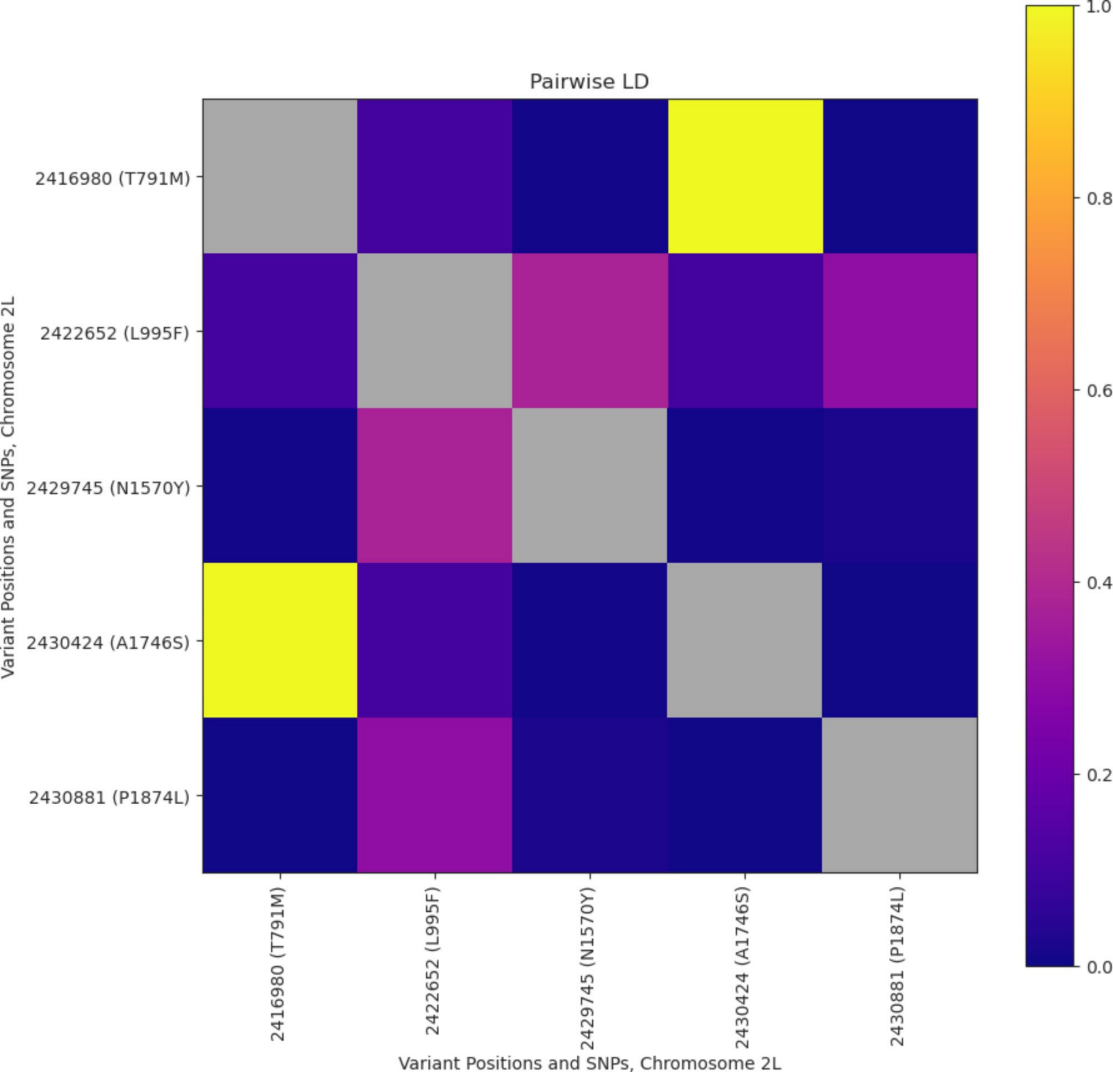
Pairwise linkage disequilibrium (Rogers and Huff) between insecticide-resistance SNPs in the *vgsc* gene. A value of 1 indicates that the two alleles are in complete linkage, which means that these two alleles are only ever found in combination with each other.

Linkage disequilibrium (LD) was calculated for each pair of variants using the method of Rogers and Huff^26^ (Fig. 1). R^2^ values are shown in Supplementary Data 1. The alternate alleles T791M and A1746S were in complete LD with an R^2^ value of 1. The alternate alleles L995F and N1570Y had an R^2^ value of 0.372, indicating that these alleles were in moderate LD. The following alternate alleles were in weak LD: T791M and L995F (R^2^ = 0.112), L995F and A1746S (R^2^ = 0.112), L995F and P1874L (R^2^ = 0.301). The T791M mutation was only ever identified in association with L995F. However, as the L995F allele was identified at a higher frequency and in samples without the presence of T791M, these two alleles were not found in high LD. Other SNP combinations showed very low or absent LD (R^2^ ≤ 0.023). Note that the sample sizes of alternate SNPs used to calculate LD were small (Table 2).

## Allele frequencies of other non-synonymous SNPs

The four genes associated with insecticide resistance that were investigated here (*vgsc*, *gste2*, *rdl* and *ace1)* were also investigated for additional non-synonymous SNPs that have not previously been associated with insecticide resistance. An additional 20 non-synonymous SNPs were identified in insecticide resistance genes (Table 3). SNPs with an allele frequency above 5% have been highlighted in bold. Odds ratios with 95% confidence intervals were calculated for these SNPs, but no significant associations were found between the resistance phenotype and these SNPs in this sample set (Supplementary Data 3).

**Table 3:** Additional non-synonymous SNPs identified in insecticide resistance genes *vgsc*, *gste2*, *rdl and ace1*. Includes the number of mosquitoes and their phenotype per SNP. SNPs with an allele frequency over 5% in the sample population are highlighted in bold. Ref = reference alt = alternate.

Chrom	Gene	Position	Ref	Alt	Amino acid Change	Resistant mosquitoes (*n* = 23)		Susceptible mosquitoes (*n* = 10)		Control mosquitoes (*n* = 9)	
						Heterozygous	Homozygous Alternate	Heterozygous	Homozygous Alternate	Heterozygous	Homozygous Alternate
2 L	*vgsc*	2,431,232	G	A	G1991E	1	0	0	0	0	0
	*vgsc*	2,431,330	A	T	T2024S	1	0	0	0	0	0
	*vgsc*	2,431,394	C	T	P2045L	0	0	0	0	1	0
3R	*gste2*	28,597,772	C	A	K215N	0	1	0	0	0	0
	*gste2*	28,597,891	C	T	E176K	2	0	1	0	1	0
	***gste2***	**28,597,956**	**G**	**C**	**T154S**	**13**	**4**	**5**	**1**	**5**	**1**
	***gste2***	**28,598,041**	**T**	**A**	**I126F**	**3**	**0**	**1**	**0**	**1**	**0**
	***gste2***	**28,598,505**	**C**	**T**	**G26S**	**2**	**0**	**1**	**0**	**0**	**0**
	*gste2*	28,598,568	C	G	V5L	1	0	0	0	1	0
	*gste2*	28,598,573	T	A	N3I	1	0	0	0	1	0
2 L	*rdl*	25,382,837	G	A	V63I	1	0	0	0	0	0
	*rdl*	25,433,554	C	T	P473S	1	0	0	0	0	0
2R	*ace1*	3,489,253	G	A	G14E	1	0	0	0	0	0
	*ace1*	3,489,358	C	G	A49G	1	0	0	0	0	0
	*ace1*	3,489,391	C	T	A60V	0	0	1	0	0	0
	***ace1***	**3,489,405**	**G**	**T**	**A65S**	**14**	**5**	**7**	**2**	**4**	**1**
	*ace1*	3,489,475	C	T	S88L	0	0	0	0	1	0
	*ace1*	3,491,883	C	T	T216I	1	0	0	0	0	0
	*ace1*	3,493,410	G	A	S648N	0	0	0	0	1	0
	*ace1*	3,493,714	G	T	G1991E	0	0	1	0	0	0

## Investigation of copy number variant (CNV) alleles associated with metabolic resistance: detection using soft-clipped reads

Copy number variants (CNVs) in genes associated with metabolic insecticide resistance were also investigated. We analysed CNVs that were previously identified by the *Anopheles gambiae* 1000 Genomes Consortium^17^. As whole genome amplification (WGA) was used to amplify the mosquito DNA, we could not reliably identify CNVs using read coverage data or quantitative PCR. Instead, we screened for these CNVs by identifying soft-clipped reads that were able to detect the CNV^17^. The investigated CNVs are listed in Supplementary Data 2 and included CNV alleles in cytochrome-P450 genes: *cyp6aa – cyp6p* region, *cyp6m – cyp6z* region and *cyp9k1*, and within the *gstu - gste* cluster region. This analysis indicated the putative presence of four CNV alleles of interest in our Bijagós mosquitoes: Cyp6aap_Dup7, Cyp6aap_Dup11, Cyp9k1_Dup12, and Gstue_Dup3. Three of these CNV alleles were identified within the susceptible mosquito population: Cyp6aap_Dup11 and Gsteu_Dup3 in one susceptible mosquito, and Cyp9k1_Dup12 in one susceptible mosquito. One CNV allele was present in one resistant mosquito, Cyp6aap_Dup7. CNV allele counts can be found in Supplementary Data 2.

## Windowed measures of differentiation and selection

The fixation index (F_ST_) is a measure of genetic differentiation between populations^6^. F_ST_ was calculated between resistant and susceptible mosquitoes over each chromosome in 1Kbp windows. This metric was used to identify regions of differentiation between the resistant and susceptible mosquitoes. F_ST_ scores were plotted along each chromosome, and significant peaks in F_ST_ were identified (Supplementary Data 1), which encompassed 51 protein-coding genes (Table 4). The top five significant F_ST_ values for each chromosome are summarised in Table 4, with more detail on F_ST_ significance criteria available in Supplementary Data 1.

**Table 4:** Genes identified within F_ST_ windows of significance, between resistant and susceptible *Anopheles gambiae* s.s. whole genome sequence data.

Chromosome	Gene	Description (Vector Base)	F_ST_ value
2L	AGAP004835	Protein coding gene, unspecified product	0.177
	AGAP029530	Protein coding gene - unspecified product	0.150
	AGAP005510	Oxysterol binding protein-like 9	0.153
	AGAP007133	Protein coding gene - unspecified product	0.140
	AGAP007576	Protein coding gene - unspecified product	0.196
2R	AGAP001535	Histone-lysine N-methyltransferase ASH1L	0.156
	AGAP001786	Protein coding gene - unspecified product	0.184
	AGAP002748	Protein kinase C	0.163
	AGAP029576	Protein coding gene – unspecified product	0.155
	AGAP003115	Protein coding gene - unspecified product	0.199
3L	AGAP010621	Glycine transporter	0.198
	AGAP029077	Protein coding gene - unspecified product	0.174
	AGAP029564	Ig-like domain-containing protein	0.293
	AGAP029790	Protein coding gene - unspecified product	0.195
	AGAP028685	DUF4806 domain-containing protein	0.195
3R	AGAP010407	Elongator complex protein 4	0.311
	AGAP010884	Protein coding gene – unspecified product	0.133
	AGAP011540	Dynein intermediate chain 2, axonemal	0.156
	AGAP029516	Protein coding gene - unspecified product	0.128
	AGAP029760	Clustered mitochondria protein homolog	0.135
X	AGAP000433	Ras-related protein Rab-39B	0.145
	AGAP029672	Protein tweety homolog	0.150
	AGAP029671	Protein coding gene - unspecified product	0.150
	AGAP000863	Lachesin	0.125
	AGAP000932	Protein coding gene - unspecified product	0.127

### Haplotype clusters

Each window of the genome with a ‘peak’ in F_ST_ was treated as a putative window of interest. For each of these windows, hierarchical clustering of haplotypes was performed to identify high frequency haplotypes which may be under directional selection, making them candidates for association with insecticide resistance. We identified clusters of ≥ 20 highly similar haplotypes in two windows of chromosome 3L (7295325–7305324) and (7296325–7306324), one window of chromosome 3R (52339701–52349700) and in 10 windows of chromosome X. There were no significant differences in the haplotypes present between the resistant and the susceptible mosquitoes (Supplementary Data 1). A larger sample size is likely necessary to reveal associations between haplotype clusters and the resistance phenotype.

## Genome wide selection scan: H12 for recent selective sweeps

**Fig. 2:**
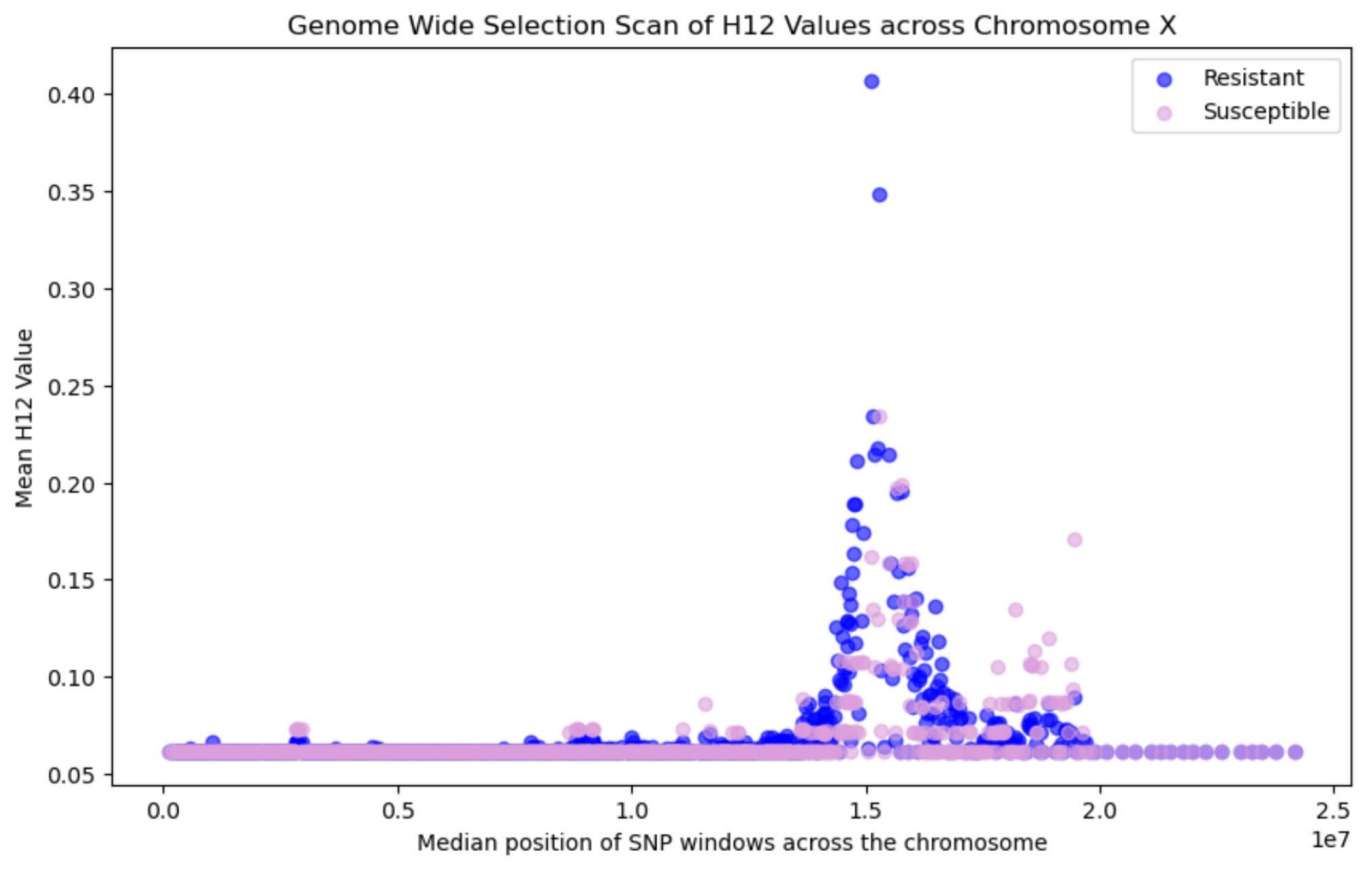
Genome Wide Selection Scan: H_12_ values across chromosome X for resistant (blue) and susceptible (pink) mosquitoes. The peak in H_12_ values indicates a selective sweep over this portion of the chromosome.

**Fig. 3:**
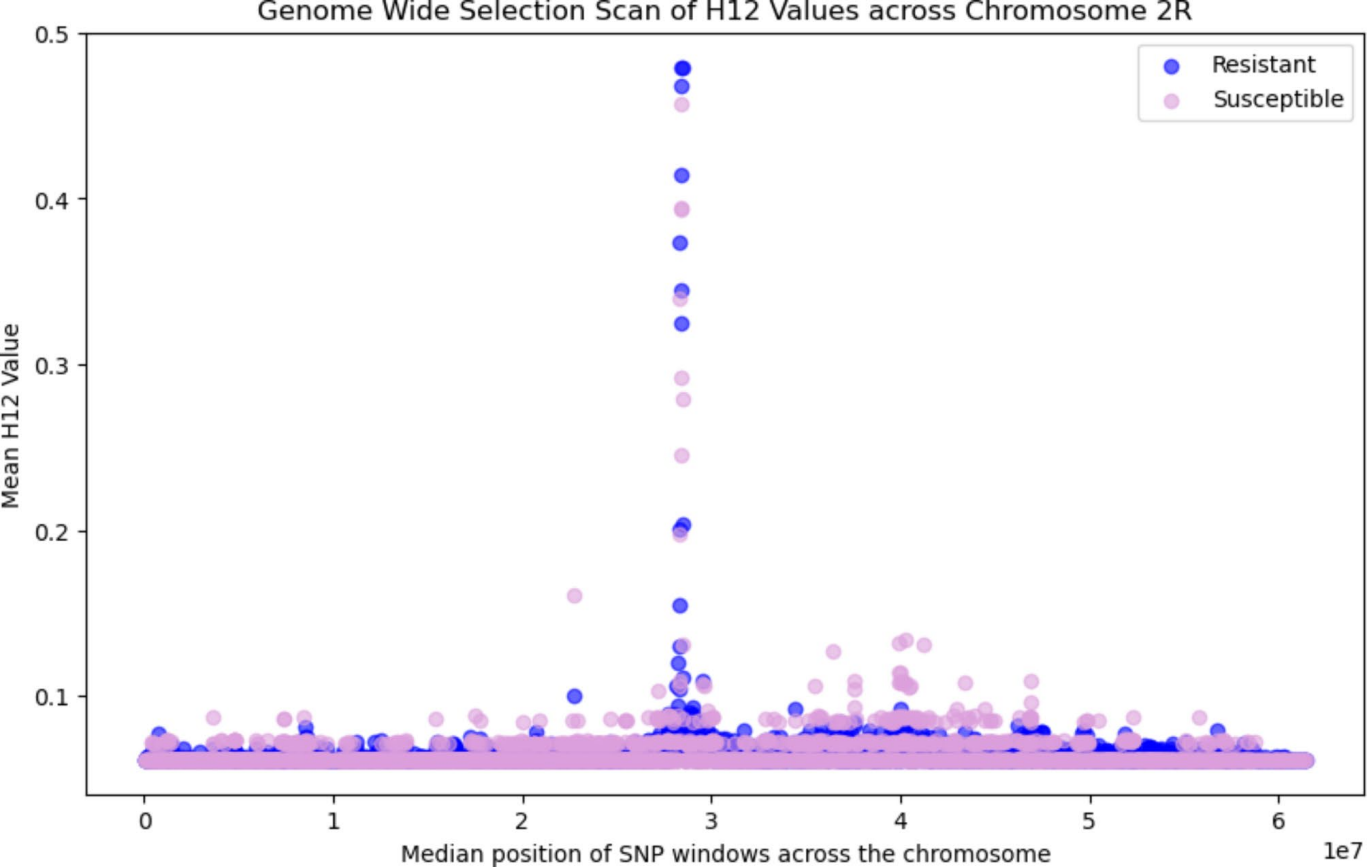
Genome Wide Selection Scan: H_12_ values across chromosome 2R for resistant (blue) and susceptible (pink) mosquitoes. The peak in H_12_ values indicates a selective sweep over this portion of the chromosome.

We calculated Garud’s H_12_ statistic in 1000 SNP windows across the genome to detect signatures of recent selective sweeps (Fig. 2)^6,27^. H_12_ is a statistic designed to identify both hard and soft selective sweeps^28^. This metric was calculated for susceptible and resistant mosquitoes. We found the signatures of two recent selective sweeps in the population; one on Chromosome X (Fig. 2) and one on Chromosome 2R (Fig. 3), but no sweeps that were unique to resistant mosquitoes. These peaks indicate selection in the mosquito population as a whole, but do not distinguish whether these sweeps are associated with resistance to deltamethrin. Selective sweeps were not identified in chromosomes 2L, 3L or 3R, where no H_12_ values greater than 0.2 were found (Supplementary Data 1).

Protein coding genes identified in the peaks of H_12_ are summarised in Supplementary Data 1. Overlapping genes were identified if they were in a 1000 SNP window with a H_12_ value above 0.2. This identified 55 different genes in chromosome X and 12 different genes in chromosome 2R. These genes included the cytochrome P450 genes *cyp9k1*,* cyp6aa1*,* cyp6aa2*,* coeae60*,* cyp6p15p*,* cyp6p3*,* cyp6p5*,* cyp6p4*,* cyp6p1*,* cyp6p2*, and *cyp6ad1.*

## Discussion

Monitoring and managing the evolution of insecticide resistance is a vital component in the prevention and elimination of vector-borne diseases^3^. Improvements in sequencing technology have enabled the generation of whole genome sequence (WGS) data for vectors on a large scale^29^. This data can be used to identify molecular markers of insecticide resistance, which can supplement the results of phenotypic bioassays. This study used WHO tube tests^5^ to investigate the presence of deltamethrin resistance in mosquitoes on Bubaque island in the Bijagós Archipelago. This was followed by WGS of *Anopheles gambiae* s.s. mosquitoes to investigate molecular markers associated with resistance.

Phenotypic bioassays revealed deltamethrin resistance in the mosquito population according to WHO guidelines^5^, and intensity testing indicated that resistance is of moderate to high intensity. These are important findings, particularly considering the reliance on pyrethroid ITNs for vector control in the Bijagós. This data can be incorporated into future decisions on which vector-based control tools to implement. Pyrethroid resistance has become widespread across malaria endemic countries^20^, and a number of updated ITN recommendations have been issued in recent years^4^. In 2017, WHO recommended the use of pyrethroid-PBO ITNs, which contain the synergist piperonyl butoxide (PBO), which inhibits cytochrome P450 metabolic enzymes within mosquitoes and enhances the potency of the pyrethroid net component^2^. This was followed in 2023 by a strong recommendation for the deployment of pyrethroid-chlorfenapyr ITNs in areas of pyrethroid resistance^4^. These pyrethroid-chlorfenapyr ITNs combine a pyrethroid with a pyrrole insecticide with a separate mode of action, increasing the efficacy of the net^4^. A third type of ITN, pyrethroid-pyriproxyfen nets, were also given a conditional recommendation by WHO for deployment instead of pyrethroid-only nets in areas of pyrethroid resistance. These nets combine a pyrethroid with the insect growth regulator pyriproxyfen, which disrupts mosquito growth and reproduction^30^. Given that this study identified resistance to pyrethroids on the most populated island of the Bijagós, these recommendations have increased relevance in the area. Future studies would benefit from conducting additional WHO intensity bioassays with 5x and 10x deltamethrin concentrations to gather more information about the intensity of resistance. In addition, future studies would benefit from synergist-insecticide bioassays to further investigate metabolic resistance in this population^31^. Furthermore, it would be informative to collect insecticide bioassay data from other islands in the archipelago, as one limitation of this study is that experiments were conducted on Bubaque island only.

WGS analysis of *Anopheles gambiae* s.s. mosquitoes revealed the presence of six SNPs that have previously been associated with, or putatively associated with, insecticide resistance. These were the *vgsc* SNPs: T791M, L995F, N1570Y, A1746S, and P1874L, and the *gste2* SNP L119V. Two mutations: *vgsc* N1570Y and *gste2* L119V, were identified in resistant mosquitoes but not in susceptible mosquitoes. Previous research has shown that the N1570Y mutation increases pyrethroid resistance levels when in combination with L995F^12^. Analysis of linkage disequilibrium identified that the T791M and the A1746S mutations were in complete linkage disequilibrium. Twenty additional non-synonymous SNPs were identified across the four insecticide resistance genes which have not previously been associated with insecticide resistance. Of these, four mutations were present at > 5% frequency, including T154S, I126F and G26S in the *vgsc* gene and A65S in *ace1.* All four of these mutations were present in both resistant and susceptible mosquitoes. One of these SNPs, T154S, was previously reported in mosquitoes from the Bijagós^19^, Guinea, Ivory Coast and the laboratory strain Kisumu originally isolated from Kenya^32^, warranting further investigation. The other SNPs at frequency of > 5% have not previously been reported in the literature to our knowledge.

In our previous study, multiplex amplicon sequencing of > 200 mosquitoes collected in 2019 identified four mutations previously associated with, or putatively associated with, insecticide resistance: *vgsc* L995F, N1570Y, A1746S and *rdl* A296G^19^. In contrast, this study did not identify the *rdl* A296G mutation. This SNP was previously identified at very low frequency, so its absence may be explained by the smaller sample set of mosquitoes used in this study^19^. Of note, the mosquitoes sequenced in this study were collected over a smaller geographical area than those collected in 2019^19^.

The presence of copy number variants (CNVs) in metabolic genes offers the possibility of elevated transcription of genes associated with enzymatic detoxification of insecticides, and has been strongly linked with insecticide resistance in *Anopheles* mosquitoes^33^. A number of CNVs of interest have previously been identified in *Anopheles gambiae* s.l^17^. Because we used WGA genomic data, we could not analyse CNVs using read coverage data or quantitative PCR. Therefore, we used a rapid screening method to indicate the likely presence of these specific CNVs through the analysis of soft clipped reads at previously identified breakpoints^17^. The only CNV allele indicated in this study that was unique to resistant mosquitoes was Cyp6aap_Dup7. This soft-clipping screen is only indicative and to confirm the presence or absence of CNVs in future studies, WGS should be conducted without prior WGA, allowing CNVs to be verified through the analysis of read coverage data. This is a small sample set of deltamethrin resistant mosquitoes, but the indicative presence of Cyp6aap_Dup7 is particularly interesting; this CNV contains two cytochrome P450 genes adjacent to the *cyp6* gene cluster, *cyp6aa1* and *cyp6aa2*, and contains part of the carboxylesterase *coeae60* gene^17^. Cyp6aap_Dup7 has been found previously in *An. gambiae* s.l. in Burkina Faso, Côte d’Ivoire, Ghana and Guinea^17^ and *cyp* gene amplifications have previously been identified in mosquitoes from mainland Guinea-Bissau^7^. Copy number of *cyp6aa1* has previously been associated with deltamethrin resistance in *An. coluzzii* in Ghana^6^, and copy number of *cyp6aa2* has been associated with deltamethrin resistance in *An. coluzzii* in Côte d’Ivoire^34^. Furthermore, transcription of c*yp6aap1* was higher in pyrethroid resistant *An. coluzzii* compared to susceptible colonies in Burkina Faso^35^, and CYP6AA1 was overexpressed in pyrethroid resistant *An. gambiae* s.s. compared to susceptible colonies in Tanzania^36^. CYP6AA1 has been shown to metabolize pyrethroids in *Drosophila melanogaster*, and modelling indicates that it should be able to bind to permethrin and deltamethrin in *An. gambiae*^14^. One limitation of our approach is that we were only able to investigate CNVs which had previously been identified by the Ag1000G project^17^, and only those that consistently identified with soft-clipped reads during our verification step. We are only able to indicate the putative presence of CNV alleles using this screening method, and other CNV alleles associated with resistance may be present in the population.

Along with investigating SNPs and CNV alleles, we analysed the WGS data for signatures of differentiation and selection. Genes identified with significant fixation index (F_ST_) scores included the *CPFL4* gene (Cuticular protein 4 from CPFL family - AGAP010905, F_ST_ = 0.122) on chromosome 3L. This gene family encodes structural cuticular proteins, for which differential expression has been associated with insecticide resistance in *An. arabiensis*^37^. We identified two selective sweeps in the *Anopheles gambiae* s.s. mosquito genomes. These sweeps are not associated with resistance in our small sample set, but they do indicate selection within the mosquito population. The selective sweep on chromosome X included the *cyp9k1* gene, which is a cytochrome P450 gene able to metabolise deltamethrin^15,16^. This gene has previously been associated with deltamethrin resistance in *An. gambiae* s.l^6^., *An. funestus*^16^ and *An. coluzzii*^38^, and has been found to be up-regulated in mosquitoes that are resistant to pyrethroids^39^ and DDT^40^. This study identified the putative presence of one CNV allele containing the *cyp9k1* gene in the Bijagós population (Cyp9k1_Dup12)^17^. The selective sweep identified in chromosome 2R overlapped with several metabolic genes associated with insecticide resistance from the *cyp6aa/cyp6p* gene cluster, including *cyp6aa1*,* cyp6aa2*,* cyp6p1*,* cyp6p2*,* cyp6p3*,* cyp6p4*,* cyp6p5*,* cyp6p15p* and *cyp6ad1*, and the carboxylesterase gene *coeae60.* Overexpression of the *coeae60* gene has previously been associated with permethrin resistance in *An. coluzzii*^41^. Multiple studies have identified associations between these genes from the Cyp6 subfamily and pyrethroid resistance; upregulation of *cyp6m2* and *cyp6p3* has been identified in pyrethroid resistant *An. gambiae*^42^ and *An. coluzzii*^38^, expression of *cyp6p3* has also been shown to have a role in carbamate resistance, and expression of *cyp6p4* has been associated with pyrethroid resistance in *An. arabiensis*^42^. Of note, the putative CNV allele that we found indicated within our resistant mosquito population (Cyp6aap_Dup7) contains the genes *cyp6aa1*,* cyp6aa2*, and part of *coeae60*^17^, associated with metabolic insecticide resistance. The indicative presence of CNV alleles and selective sweeps over metabolic genes indicates that metabolic resistance is a key component of insecticide resistance on the Archipelago. However, synergist assays are required to further understand the extent of metabolic resistance.

This study provides evidence of deltamethrin resistance and the first whole genome sequence data analysis of *An. gambiae* s.s. mosquitoes from the Bijagós Archipelago. Our genome-wide selection scan using H_12_ has identified two selective sweeps, both of which contain metabolic resistance genes that have previously been associated with metabolic resistance to pyrethroids. These selective sweeps indicate that the use of pyrethroid-based ITNs in the Bijagós has likely resulted in selection in metabolic genes. Future studies should include synergist-insecticide bioassays to investigate the presence of metabolic resistance in this mosquito population. The small sample sizes in this study have made drawing statistically significant conclusions between the presence of SNPs, sweeps, haplotype clusters and the resistance phenotype difficult. However, our work provides a baseline for future studies with larger sample sizes to measure robust statistical significance. Furthermore, this study provides mortality data for deltamethrin resistance which can contribute towards evidence-based decision making in the selection of future vector control measures.

### Methods

#### Mosquito sampling and insecticide resistance assays

Mosquito larvae were collected by dipping in three in-land pools close to Bubaque village on Bubaque island in November 2022. All larvae were collected in vials and reared in larval trays using their native water where possible. Upon eclosion, adult mosquitoes were maintained in 35 × 35 × 35 cm insect rearing cages (BugDorm, Taichung, Taiwan) with 10% sucrose solution. WHO tube tests were conducted with non-bloodfed females between 3 and 5 days old as per WHO guidelines^5^. Susceptibility to deltamethrin was measured at discriminating doses of 0.05% and at 5x concentration (0.25%) using deltamethrin-treated paper, which was procured from Universiti Sains Malaysia, Penang, Malaysia. Deltamethrin 0.05% concentration tests were conducted over 5 replicates, with an average of 20 mosquitoes introduced per tube. Deltamethrin 0.25% concentration tests were conducted over 3 replicates, with an average of 15 mosquitoes introduced per tube. Control tests were conducted using control test papers treated with oil as opposed to insecticide, which were procured from Universiti Sains Malaysia, Penang.

### DNA extraction and species identification

DNA was extracted using Dynabeads™ (ThermoFisher Scientific Inc), following the standard protocol. Species identification was conducted using the Bass (2008) qPCR protocol^43^ to distinguish *An. gambiae (An. gambiae* s.s, *An. coluzzii* and *An. gambiae – An. coluzzii* hybrids) from *An. melas.* The Santolamazza (2008) SINE200 PCR^44^ was then used to distinguish between *An. gambiae* s.s, *An. coluzzii* and *An. gambiae – An. coluzzii* hybrids. DNA was quantified using the Qubit dsDNA HS Kit (Thermo Fisher Scientific Inc).

### Whole genome sequencing

Mosquitoes identified as *Anopheles gambiae* s.s. were selected for whole genome sequencing. A small quantity of genetic material was available for whole genome sequencing, so samples were processed using Whole Genome Amplification (WGA) prior to sequencing. WGA was conducted using the REPLI-g^®^ mini kit (Qiagen) following the standard protocol. This included 23 resistant, 10 susceptible and 9 control mosquitoes. Control mosquitoes were used in phenotypic bioassays as control mosquitoes for survival, so the resistance phenotype for controls is unknown. DNA was sequenced at Eurofins Genomics GmbH, Germany, using the Illumina Novaseq 6000 (2 × 150 bp paired-end read configuration).

### Bioinformatic analysis

Raw FastQ files were trimmed using *trimmomatic* software (version 0.39, using the parameters LEADING:3 TRAILING:3 SLIDINGWINDOW:4:20 MINLEN:36) to remove poor quality sequences^45^. Trimmed data was aligned to the *Anopheles gambiae* reference genome (Anopheles_gambiae.AgamP4.dna.toplevel.fa), using *bwa-mem* software (version 0.7.17-r1188, default parameters) to produce a BAM file for each sample. The *samtools* (version 1.12) functions fixmate and markdup were applied to the resulting BAM files^46^. SNPs were called using *GATK*’s HaplotypeCaller (version 4.1.4.1) using the option -ERC GVCF. Validated VCFs were merged into a database using *GATK*’s GenomicsDBImport function, and a combined VCF was created using *GATK*’s GenotypeGVCFs function^47^. This combined VCF was filtered for high quality variants using *bcftools* (version 1.17)^46^. Samples were retained if 40% of the genome had > 10-fold coverage. *GATK* VariantFiltration was then used to retain SNPs with high quality following *GATK* filter recommendations: QD > 5.0, QUAL > 30.0, SOR < 3.0, FS < 60.0, MQ > 40.0, MQRankSum > -12.5 and ReadPosRankSum > -8.0. *Bcftools* was used to retain SNPs with DP > 5 and QQ > 20, and to remove variants with a high proportion of missing genotypes (> 20%). The filtered VCF was phased using *beagle* (version 5.2)^48^.

Linkage disequilibrium (LD) was calculated using the method of Rogers and Huff^26^, using the *allel.rogers_huff_r* function in scikit-allel (https://scikit-allel.readthedocs.io/en/stable/), to provide an R^2^ value for each combination of target SNPs in the *vgsc* gene.

Due to low sample availability in this study, samples underwent whole genome amplification (WGA) prior to whole genome sequencing. Therefore, we could not reliably identify CNVs using read coverage data, as coverage can be distorted by WGA. Instead, we used a rapid screening method to investigate the presence of specific CNV alleles of interest in the Bijagós mosquitoes by identifying soft clipping. This involved computing the proportion of reads that had been soft-clipped at CNV breakpoints previously described by Lucas et al. (2019)^17^. In doing so, we screened for 40 of these previously identified CNV alleles by analysing the proportion of soft-clipped reads in mapped reads with mapping quality score ≥ 10 (Supplementary Data 2). Normalised clipping was computed from BAM files as the proportion of reads at each position that had been soft clipped. Soft-clipping screening was conducted for a ‘verification set’ of samples available from The *Anopheles gambiae* 1000 Genomes Project phase 3 data resource, accessed via the MalariaGEN Ag3 API client, for which CNV data is available. Screening for soft-clipping at known breakpoints in each CNV of interest^17^ was able to correctly identify the presence of CNVs, indicated by a high proportion of soft clipped reads present at the start, end, or both ends of the known CNV. A threshold of proportion soft clipped was set at 19.5% from the soft-clipping proportions computed for the verification set of samples (Supplementary Data 2).

F_ST_ was calculated between resistant and susceptible mosquitoes for non-overlapping 1 Kbp windows of the genome using the *allel.windowed_patterson_fst* function in scikit-allel. As described in Lucas et al. (2023)^6^, peaks in F_ST_ could have been caused by extended haplotype homozygosity in a region due to a selective sweep, even if that sweep were not due to phenotype, because the non-independence of SNPs within that window would lead to increased F_ST_ variance compared to other regions of the genome^6^. To identify peaks that were associated with phenotype, we performed 200 simulations in which the phenotype of the mosquitoes was randomly permuted and F_ST_ was recalculated. F_ST_ windows of interest were then kept if their F_ST_ value was higher than the 99th centile of these simulations. Additionally, we only kept F_ST_ windows of interest if the F_ST_ value was higher than three times the minimum distribution, as in Lucas et al. 2023^6^, to reduce noise.

Haplotype clusters were determined by hierarchical clustering on pairwise genetic distance between haplotypes. Dendrograms were generated using the *scipy_cluster_hierarchy* function in SciPy (version 1.11.1), linkage method = single and metric = hamming. Clusters with > 20 similar haplotypes were included in Supplementary Data 1.

To detect regions of the genome undergoing selective sweeps, which may be due to selection pressure for insecticide resistance, we performed a genome wide selection scan using Garud’s H_12_ statistic^28,29^. Garud’s H_12_ was calculated using the *garuds_h* function in scikit-allel (https://scikit-allel.readthedocs.io/en/stable/), using phased biallelic SNPs in windows of 1000 SNPs. 200 Iterations of H_12_ were computed for samples, and the mean value for each 1Kbp window of SNPs was plotted.

## Electronic supplementary material

Below is the link to the electronic supplementary material.

Supplementary Material 1

Supplementary Material 2

Supplementary Material 3

## Data Availability

The processed datasets are available at ENA Project PRJEB71957, https://www.ebi.ac.uk/ena/browser/view/PRJEB71957. All code used to analyse the data can be found at https://github.com/sophiemoss/anopheles_popgen.
