# Supplementary material for: Phenotypic evidence of deltamethrin resistance and identification of selective sweeps in Anopheles mosquitoes on the Bijagós Archipelago, Guinea-Bissau
Source: Sci Rep. 2024 Oct 1;14:22840. doi: 10.1038/s41598-024-73996-3 (PMC11445403; doi:10.1038/s41598-024-73996-3)
Supplement: Supplementary file 2 — Supplementary Material 2 [file 41598_2024_73996_MOESM2_ESM.docx]

Supplementary Data 1

Supplementary Table 1: Insecticide resistance SNPs of interest

| **Position** | **Chromosome** | **SNP** | **Gene** |
| --- | --- | --- | --- |
| 2390177 | 2L | R254K | *vgsc* |
| 2391228 | 2L | V402L | *vgsc* |
| 2399997 | 2L | D466H | *vgsc* |
| 2400071 | 2L | M490I | *vgsc* |
| 2402466 | 2L | G531V | *vgsc* |
| 2407967 | 2L | Q697P | *vgsc* |
| 2416980 | 2L | T791M | *vgsc* |
| 2422651 | 2L | L995S | *vgsc* |
| 2422652 | 2L | L995F | *vgsc* |
| 2429556 | 2L | V1507I | *vgsc* |
| 2429617 | 2L | I1527T | *vgsc* |
| 2429745 | 2L | N1570Y | *vgsc* |
| 2429897 | 2L | E1597G | *vgsc* |
| 2429915 | 2L | K1603T | *vgsc* |
| 2430424 | 2L | A1746S | *vgsc* |
| 2430817 | 2L | V1853I | *vgsc* |
| 2430863 | 2L | I1868T | *vgsc* |
| 2430880 | 2L | P1874S | *vgsc* |
| 2430881 | 2L | P1874L | *vgsc* |
| 2431061 | 2L | A1934V | *vgsc* |
| 2431079 | 2L | I1940T | *vgsc* |
| 28598166 | 3R | I114T | *gste2* |
| 28598057 | 3R | F120L | *gste2* |
| 28598062 | 3R | L119V | *gste2* |
| 25429236 | 2L | A296G | *rdl* |
| 25429235 | 2L | A296S | *rdl* |
| 3492074 | 2R | G280S* | *ace1* |

Supplementary Table 2: Candidate resistance SNP genotypes in Anopheles gambiae s.s. mosquitoes. Table values indicate the number of mosquitoes with each genotype for each SNP (homozygous reference, heterozygous, or homozygous alternate). ‘No call’ columns indicate the number of samples where the data available for this position was not adequate to make a reliable genotype call.

| Chromosome | Gene | Position | SNP | Resistant mosquitoes (n=23) | | | | Susceptible mosquitoes (n=10) | | | | Control  mosquitoes (n=9) | | | |
| --- | --- | --- | --- | --- | --- | --- | --- | --- | --- | --- | --- | --- | --- | --- | --- |
|  |  |  |  | Homozygous reference (0/0) | Heterozygous (0/1) | Homozygous alternate (1/1) | No call | Homozygous reference (0/0) | Heterozygous (0/1) | Homozygous alternate (1/1) | No call | Homozygous reference (0/0) | Heterozygous (0/1) | Homozygous alternate (1/1) | No call |
| 2L | *vgsc* | 2416980 | T791M | 18 | 0 | 0 | 5 | 8 | 1 | 0 | 1 | 8 | 1 | 0 | 0 |
| 2L | *vgsc* | 2422652 | L995F | 15 | 7 | 0 | 1 | 7 | 3 | 0 | 0 | 6 | 3 | 0 | 0 |
| 2L | *vgsc* | 2429745 | N1570Y | 18 | 5 | 0 | 0 | 10 | 0 | 0 | 0 | 7 | 1 | 0 | 1 |
| 2L | *vgsc* | 2430424 | A1746S | 23 | 0 | 0 | 0 | 9 | 1 | 0 | 0 | 7 | 1 | 0 | 1 |
| 2L | *vgsc* | 2430881 | P1874L | 21 | 2 | 0 | 0 | 8 | 2 | 0 | 0 | 7 | 1 | 0 | 1 |
| 3R | *gste2* | 28598062 | L119V | 19 | 3 | 0 | 1 | 9 | 0 | 0 | 1 | 9 | 0 | 0 | 0 |

Supplementary Table 3: Linkage disequilibrium R2 values for pairwise combinations of insecticide resistance mutations in vgsc

| R^2^ value | 2416980 (T791M) | 2422652 (L995F) | 2429745 (N1570Y) | 2430424 (A1746S) | 2430881 (P1874L) |
| --- | --- | --- | --- | --- | --- |
| 2416980 (T791M) | 0.000 | 0.112 | 0.008 | 1.000 | 0.007 |
| 2422652 (L995F) | 0.112 | 0.000 | 0.372 | 0.112 | 0.301 |
| 2429745 (N1570Y) | 0.008 | 0.372 | 0.000 | 0.008 | 0.023 |
| 2430424 (A1746S) | 1.000 | 0.112 | 0.008 | 0.000 | 0.007 |
| 2430881 (P1874L) | 0.007 | 0.301 | 0.023 | 0.007 | 0.000 |

**Supplementary Information: F_ST_**

F_ST_ was calculated between resistant and susceptible mosquitoes. This was calculated over 1000bp windows for each chromosome using the scikit-allel, *allel.windowed_patterson_fst* function. F_ST_ values can be seen plotted in blue. To identify significant F_ST_ values we followed the methodology in Lucas *et al* (2023)^2^: we found the difference between the smallest F_ST_ value and the mode of the distribution, and considered an outlier to be any value more than three times this distance away from the mode. We also calculated 200 permutations of F_ST,_ which can be seen plotted in grey. F_ST_ values were considered significant if they were greater than the 99^th^ percentile of the 200 permutations for that window. 1000bp windows with significant F_ST_ values are highlighted with dark blue dots.


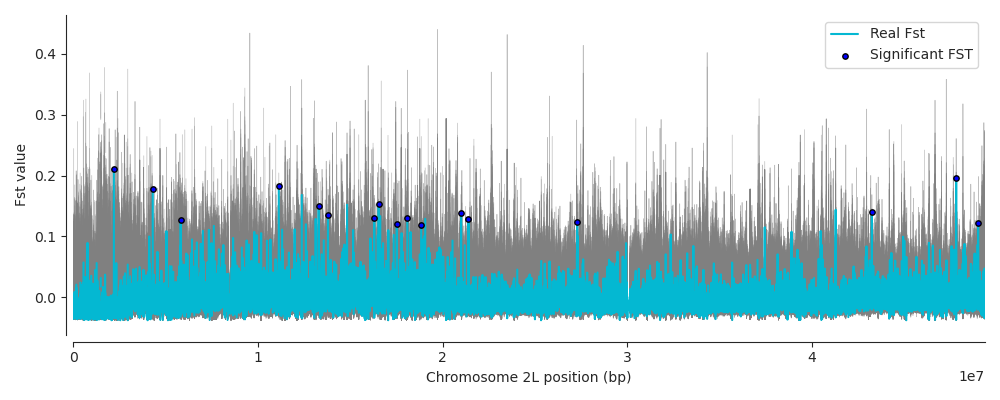


Supplementary Figure 1: FST values between resistant and susceptible mosquitoes across Chromosome 2L

There were 16 provisional windows of interest identified on chromosome 2L, with F_ST_ values greater than the 99^th^ percentile of 200 permuted F**_ST_** values.

Supplementary Table 4: Provisional windows of interest identified on chromosome 2L, where significant FST values were calculated between resistant and susceptible mosquitoes.

| **Window (chromosome 2L)** | **‘Real’ F_ST_ Value** | **99th Percentile of 200 permutated F_ST_ values** | **Feature(s) in overlapping window** |
| --- | --- | --- | --- |
| 2194206 -2195205 | 0.211 | 0.107 | - |
| 4299206 - 4300205 | 0.177 | 0.074 | AGAP004835 – protein coding gene, unspecified product |
| 5811206 - 5812205 | 0.127 | 0.084 | - |
| 11122206 - 11123205 | 0.183 | 0.176 | - |
| 13283206 - 13284205 | 0.150 | 0.101 | AGAP029530 - protein coding gene - unspecified product |
| 13785206 - 13786205 | 0.135 | 0.132 | - |
| 16287206 - 16288205 | 0.130 | 0.118 | AGAP005505 - RNA-binding protein 45 |
| 16529206 - 16530205 | 0.153 | 0.085 | AGAP005510 - oxysterol binding protein-like 9 |
| 17512206 - 17513205 | 0.120 | 0.102 | - |
| 18079206 - 18080205 | 0.129 | 0.090 | AGAP005637 - aldehyde oxidase |
| 18832206 - 18833205 | 0.119 | 0.064 | - |
| 20991206 - 20992205 | 0.139 | 0.103 | - |
| 21390206 - 21391205 | 0.129 | 0.080 | - |
| 27276206 - 27277205 | 0.123 | 0.086 | AGAP029236 - putative metabotropic glutamate receptor 1 |
| 43230206 - 43231205 | 0.140 | 0.122 | AGAP007133 – protein coding gene - unspecified product |
| 47794206 - 47795205 | 0.196 | 0.168 | AGAP007576 – protein coding gene - unspecified product |
| 48981206 - 48982205 | 0.121 | 0.048 | AGAP029465 – protein coding gene -unspecified product |

**Chromosome 2R**


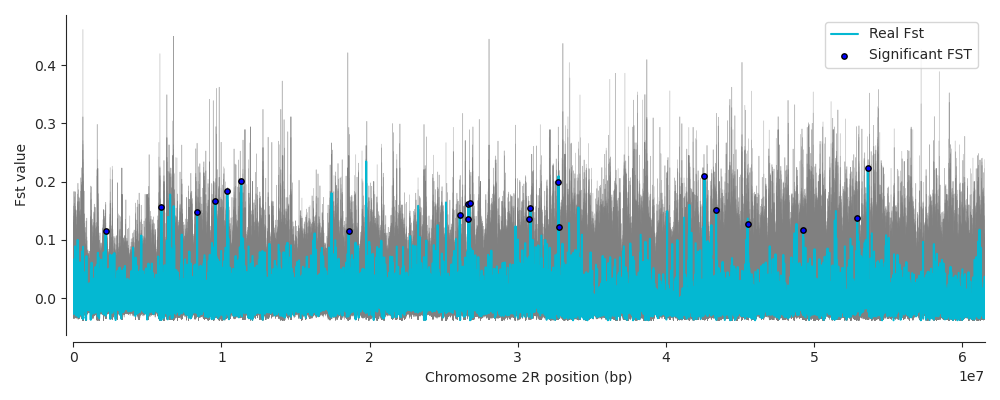


Supplementary Figure 2: FST values between resistant and susceptible mosquitoes across Chromosome 2R

There were 21 provisional windows of interest identified on chromosome 2R, with F_ST_ values greater than the 99^th^ percentile of 200 permuted F**_ST_** values.

Supplementary Table 5: Provisional windows of interest identified on chromosome 2R, where significant FST values were calculated between resistant and susceptible mosquitoes.

| **Window (chromosome 2R)** | **‘Real’ F_ST_ Value** | **99th Percentile of 200 permutated F_ST_ values** | **Feature(s) in overlapping window** |
| --- | --- | --- | --- |
| 2178086 – 2179085 | 0.116 | 0.088 | AGAP001273 – karyopherin (importin) alpha 4 |
| 5946086 -5947085 | 0.156 | 0.082 | AGAP001535 – histone-lysine N-methyltransferase ASH1L |
| 8353086 – 8354085 | 0.148 | 0.116 | AGAP001683 – calcium/calmodulin-dependent serine protein kinase |
| 9578086 – 9579085 | 0.166 | 0.074 | - |
| 10394086 – 10395085 | 0.184 | 0.112 | AGAP001786 – protein coding gene – unspecified product |
| 11333086 – 11334085 | 0.201 | 0.085 | - |
| 18600086 – 18601085 | 0.115 | 0.049 | AGAP002288 -hydroxymethylglutaryl-CoA reductase (NADPH) |
| 26085086 – 26086085 | 0.142 | 0.121 | AGAP002748 -protein kinase C |
| 26645086 – 26646085 | 0.161 | 0.046 | AGAP002748 -protein kinase C |
| 26649086 – 26650085 | 0.137 | 0.052 | AGAP002748 -protein kinase C |
| 26754086 – 26755085 | 0.163 | 0.115 | AGAP002748 -protein kinase C |
| 30763086 – 30764085 | 0.136 | 0.128 | AGAP002997 – tumor necrosis factor alpha-induced protein 8-like protein |
| 30859086 – 30860085 | 0.155 | 0.089 | AGAP029576 – protein coding gene – unspecified product |
| 32748086 – 32749085 | 0.199 | 0.120 | AGAP003115 – protein coding gene – unspecified product |
| 32755086 – 32756085 | 0.123 | 0.073 | - |
| 42603086 – 42604085 | 0.209 | 0.082 | - |
| 43388086 – 43389085 | 0.151 | 0.096 | AGAP003795 – protein coding gene – unspecified productAGAP003796 – cyclin-dependent kinases regulatory subunit 1 |
| 45526086 – 45527085 | 0.127 | 0.091 | - |
| 49260086 – 49261085 | 0.116 | 0.109 | AGAP004059 – Ca-activated cl channel protein |
| 52913086 – 52914085 | 0.138 | 0.071 | AGAP004232 – pellino |
| 53629086 – 53630085 | 0.223 | 0.178 | - |

**Chromosome 3L**

**
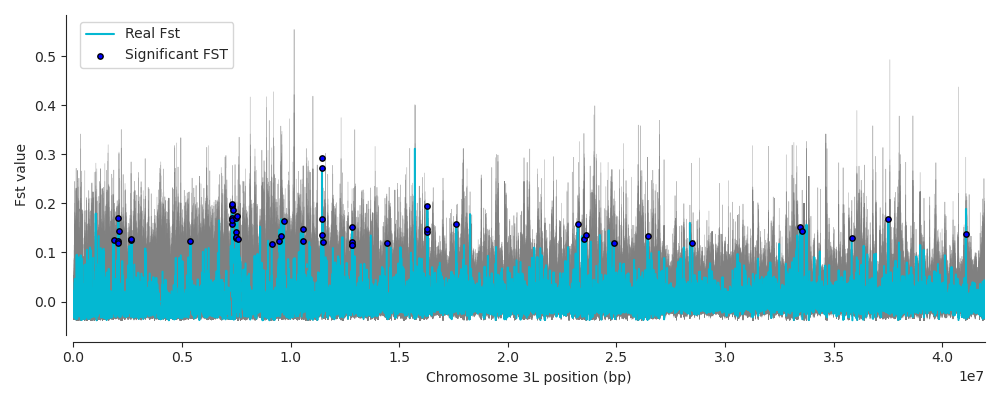
**

Supplementary Figure 3: FST values between resistant and susceptible mosquitoes across Chromosome 3L

There were 51 provisional windows of interest identified on chromosome 3L, with F_ST_ values greater than the 99^th^ percentile of 200 permuted F**_ST_** values.

Supplementary Table 6: Provisional windows of interest identified on chromosome 3L, where significant FST values were calculated between resistant and susceptible mosquitoes.

| **Window (chromosome 3L)** | **‘Real’ F_ST_ Value** | **99th Percentile of 200 permutated F_ST_ values** | **Feature(s) in overlapping window** |
| --- | --- | --- | --- |
| 1849825 - 1850824 | 0.126 | 0.085 | - |
| 2056825 - 2057824 | 0.123 | 0.119 | - |
| 2066825 - 2067824 | 0.170 | 0.099 | - |
| 2068825 - 2069824 | 0.119 | 0.100 | - |
| 2078825 - 2079824 | 0.144 | 0.128 | - |
| 2659825 - 2660824 | 0.125 | 0.081 | - |
| 2675825 - 2676824 | 0.127 | 0.111 | - |
| 5379825 - 5380824 | 0.123 | 0.113 | - |
| 7299825 - 7300824 | 0.171 | 0.119 | - |
| 7300825 - 7301824 | 0.195 | 0.136 | - |
| 7322825 - 7323824 | 0.158 | 0.152 | AGAP010621 **-**glycine transporter |
| 7323825 - 7324824 | 0.198 | 0.152 | AGAP010621 **-**glycine transporter |
| 7324825 - 7325824 | 0.166 | 0.165 | AGAP010621 **-**glycine transporter |
| 7328825 - 7329824 | 0.186 | 0.179 | - |
| 7483825 - 7484824 | 0.141 | 0.072 | - |
| 7484825 - 7485824 | 0.130 | 0.079 | - |
| 7485825 - 7486824 | 0.132 | 0.062 | - |
| 7486825 - 7487824 | 0.171 | 0.098 | - |
| 7553825 - 7554824 | 0.174 | 0.155 | AGAP029077 – protein coding - unspecified product |
| 7597825 - 7598824 | 0.128 | 0.086 | - |
| 9128825 - 9129824 | 0.116 | 0.109 | AGAP010737 – protein coding - unspecified product |
| 9444825 - 9445824 | 0.124 | 0.099 | - |
| 9554825 - 9555824 | 0.134 | 0.111 | - |
| 9696825 - 9697824 | 0.164 | 0.129 | AGAP029992 – protein coding - unspecified product |
| 10576825 -10577824 | 0.147 | 0.083 | AGAP010800 -nuclear protein NHN1 |
| 10579825 - 10580824 | 0.124 | 0.067 | AGAP010800 -nuclear protein NHN1 |
| 11424825 - 11425824 | 0.135 | 0.089 | AGAP029564 - Ig-like domain-containing protein |
| 11428825 - 11429824 | 0.293 | 0.244 | AGAP029564 - Ig-like domain-containing protein |
| 11444825 - 11445824 | 0.271 | 0.188 | AGAP029564 - Ig-like domain-containing protein |
| 11465825 - 11466824 | 0.167 | 0.165 | - |
| 11475825 - 11476824 | 0.122 | 0.087 | - |
| 12825825 - 12826824 | 0.115 | 0.071 | - |
| 12829825 - 12830824 | 0.122 | 0.113 | AGAP010905 - cuticular protein 4 from CPFL family |
| 12830825 - 12831824 | 0.152 | 0.130 |  |
| 12845825 - 12846824 | 0.114 | 0.078 |  |
| 14423825 - 14424824 | 0.120 | 0.115 | AGAP010978 - nuclear pore complex protein Nup62 |
| 16288825 - 16289824 | 0.141 | 0.114 | AGAP029789 - DUF4806 domain-containing proteinAGAP029790 – protein coding gene - unspecified product |
| 16290825 - 16291824 | 0.195 | 0.119 | AGAP029790 – protein coding gene - unspecified product  AGAP028685 - DUF4806 domain-containing protein |
| 16296825 - 16297824 | 0.148 | 0.094 | AGAP028688 – protein coding gene - unspecified product |
| 17622825 - 17623824 | 0.159 | 0.129 | - |
| 23222825 - 23223824 | 0.158 | 0.144 | - |
| 23501825 - 23502824 | 0.128 | 0.103 | - |
| 23591825 - 23592824 | 0.137 | 0.079 | AGAP011395 - patched 1 |
| 24875825 - 24876824 | 0.119 | 0.115 | - |
| 26433825 - 26434824 | 0.133 | 0.067 | AGAP011520 - CS domain-containing protein |
| 28495825 - 28496824 | 0.119 | 0.059 | - |
| 33442825 - 33443824 | 0.151 | 0.081 | - |
| 33547825 - 33548824 | 0.143 | 0.084 | - |
| 35824825 - 35825824 | 0.129 | 0.105 | AGAP011980 – protein coding gene - unspecified product |
| 37509825 - 37510824 | 0.168 | 0.124 | AGAP012087 - 7SK snRNA methylphosphate capping enzyme  AGAP012088 - stromal membrane-associated protein |
| 41074825 - 41075824 | 0.138 | 0.091 | AGAP012358 - ATP-dependent RNA helicase DDX52/ROK1 |

**Chromosome 3R**


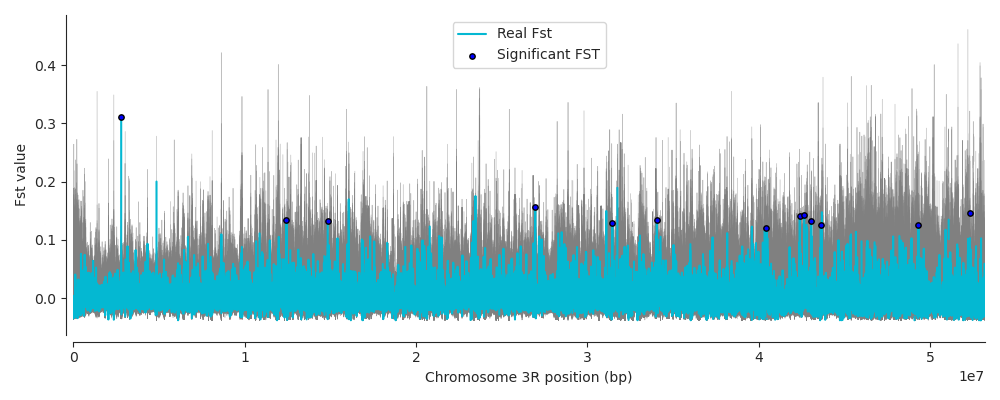


Supplementary Figure 4: FST values between resistant and susceptible mosquitoes across Chromosome 3R

There were 13 provisional windows of interest identified on chromosome 3R, with F_ST_ values greater than the 99^th^ percentile of 200 permuted F**_ST_** values.

Supplementary Table 7: Provisional windows of interest identified on chromosome 3R, where significant FST values were calculated between resistant and susceptible mosquitoes.

| **Window (chromosome 3R)** | **‘Real’ F_ST_ Value** | **99th Percentile of 200 permutated F_ST_ values** | **Feature(s) in overlapping window** |
| --- | --- | --- | --- |
| 2791201 - 2792200 | 0.311 | 0.107 | AGAP010407 - Elongator complex protein 4 |
| 12436201 - 12437200 | 0.133 | 0.112 | AGAP010884 – protein coding gene – unspecified product |
| 14843201 - 14844200 | 0.131 | 0.078 | - |
| 26950201 - 26951200 | 0.156 | 0.101 | AGAP011540 - dynein intermediate chain 2, axonemal |
| 31449201 - 31450200 | 0.128 | 0.108 | AGAP029516 – protein coding gene - unspecified product |
| 34047201 - 34048200 | 0.135 | 0.085 | AGAP029760 - clustered mitochondria protein homolog |
| 40445201 - 40446200 | 0.120 | 0.099 | - |
| 42389201 - 42390200 | 0.141 | 0.086 | - |
| 42641201 - 42642200 | 0.143 | 0.095 | - |
| 43054201 - 43055200 | 0.132 | 0.078 | - |
| 43618201 - 43619200 | 0.126 | 0.098 | - |
| 49311201 - 49312200 | 0.125 | 0.055 | - |
| 52344201 - 52345200 | 0.146 | 0.140 | - |

**Chromosome X**

**
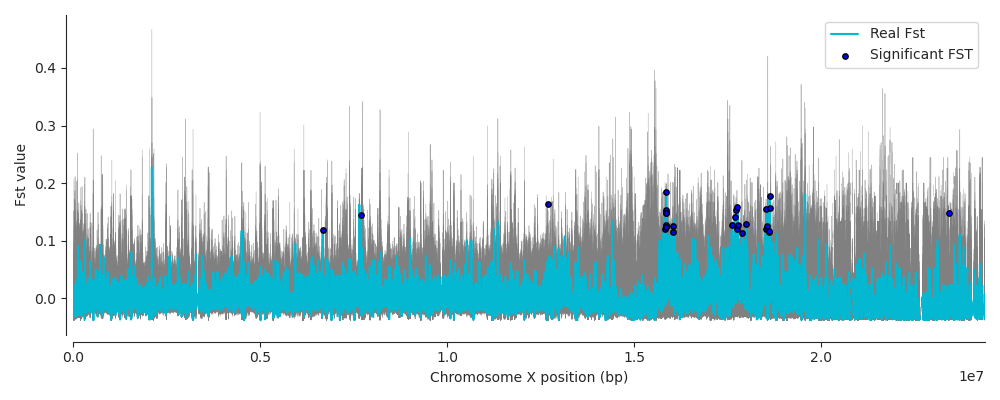
**

Supplementary Figure 5: FST values between resistant and susceptible mosquitoes across Chromosome X

There were 30 provisional windows of interest identified on chromosome X, with F_ST_ values greater than the 99^th^ percentile of 200 permuted F**_ST_** values.

Supplementary Table 8: Provisional windows of interest identified on chromosome X, where significant FST values were calculated between resistant and susceptible mosquitoes.

| **Window (chromosome X)** | **‘Real’ F_ST_ Value** | **99th Percentile of 200 permutated F_ST_ values** | **Feature(s) in overlapping window** |
| --- | --- | --- | --- |
| 6676973 - 6677972 | 0.119 | 0.072 | - |
| 7695973 - 7696972 | 0.145 | 0.112 | AGAP000433 - Ras-related protein Rab-39B |
| 12708973 - 12709972 | 0.164 | 0.090 | - |
| 15836973 - 15837972 | 0.121 | 0.095 | AGAP000859 – protein coding – unspecified product |
| 15842973 - 15843972 | 0.125 | 0.094 | AGAP029672 - protein tweety homolog |
| 15843973 - 15844972 | 0.128 | 0.112 | AGAP029672 - protein tweety homolog |
| 15846973 - 15847972 | 0.150 | 0.104 | AGAP029672 - protein tweety homolog AGAP029671 – protein coding gene - unspecified product |
| 15854973 - 15855972 | 0.148 | 0.112 | - |
| 15855973 - 15856972 | 0.154 | 0.121 | - |
| 15856973 - 15857972 | 0.185 | 0.165 | - |
| 15857973 - 15858972 | 0.149 | 0.120 | - |
| 15867973 - 15868972 | 0.124 | 0.117 | AGAP000861- protein coding gene - unspecified product |
| 16030973 - 16031972 | 0.125 | 0.105 | AGAP000863 – lachesin |
| 16043973 - 16044972 | 0.115 | 0.081 | - |
| 17620973 - 17621972 | 0.127 | 0.044 | AGAP000932 - unspecified product |
| 17703973 - 17704972 | 0.141 | 0.062 | - |
| 17738973 - 17739972 | 0.154 | 0.051 | - |
| 17745973 - 17746972 | 0.121 | 0.069 | - |
| 17766973 - 17767972 | 0.158 | 0.070 | - |
| 17772973 - 17773972 | 0.127 | 0.078 | - |
| 17900973 - 17901972 | 0.114 | 0.098 | - |
| 17999973 - 18000972 | 0.130 | 0.073 | - |
| 18523973 - 18524972 | 0.120 | 0.105 | - |
| 18531973 - 18532972 | 0.155 | 0.146 | - |
| 18567973 - 18568972 | 0.125 | 0.075 | - |
| 18621973 - 18622972 | 0.115 | 0.060 | - |
| 18623973 - 18624972 | 0.118 | 0.068 | - |
| 18624973 - 18625972 | 0.157 | 0.079 | - |
| 18627973 - 18628972 | 0.178 | 0.058 | - |
| 23417973 - 23418972 | 0.147 | 0.089 | - |

**Haplotype association**

We investigated the presence of swept haplotypes within each putative window of interest identified during the F_ST_ analysis. Hierarchical clustering was performed on haplotypes in each window of interest using pairwise genetic distances between haplotypes. This hierarchical clustering identified clusters of > 20 haplotypes that are highly similar to each other, which we refer to as ‘haplotype clusters’. Dendrograms were generated for each window of interest and the tree was cut at 1% of the maximum distance identified for each window. Dendrograms and genomic positions are included below. Genomic positions given are relative to each chromosome. Associations between haplotype clusters and insecticide resistance phenotype were explored. Mosquitoes are diploid and so have two haplotypes. For each haplotype cluster, mosquitoes were given a score of 2 if both of their haplotypes matched with a haplotype in the cluster, a score of 1 if 1 of their haplotypes matched with a haplotype in the cluster, or a score of 0 if neither of their haplotypes matched with haplotypes in the cluster. These scores were then aggregated by phenotype for each window and can be seen in the tables below. Sample sizes were not large enough to compute robust regression models to determine if there was a significant association between these haplotype clusters and resistance phenotype. However, indicative analysis does not reveal any of these haplotype clusters to be shared by a majority of resistant mosquitoes (see tables below). We conclude that a larger sample size is required to reveal associations between haplotype clusters and the resistance haplotype.

**Chromosome 3L**

Supplementary Table 9: Window 7295325 – 7305324 included one cluster of ≥20 haplotypes.

| **Window 7295325 – 7305324 haplotype cluster** | | |
| --- | --- | --- |
| Phenotype | Score | % of mosquito haplotypes |
| Resistant | 11 | 11/46 (23.9%) |
| Susceptible | 5 | 5/20 (25.0%) |
| Control | 4 | 4/18 (22.2%) |


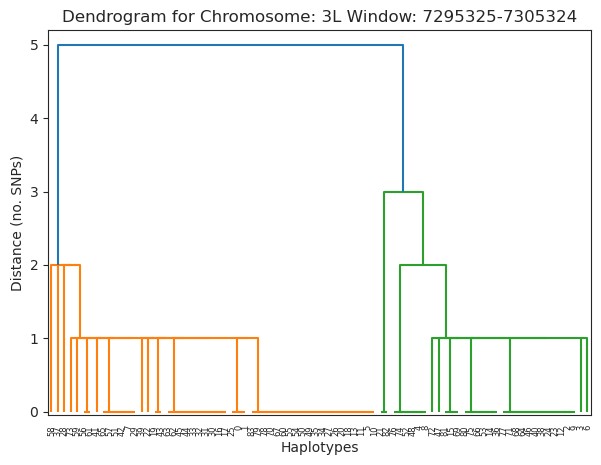


Supplementary Figure 6: Dendrogram for Chromosome 3L, Window 7295325 - 7305324

Supplementary Table 10: Window 7296325-7306324 included one cluster of ≥ 20 haplotypes.

| **Window 7296325-7306324 haplotype cluster** | | |
| --- | --- | --- |
| Phenotype | Score | % of mosquito haplotypes |
| Resistant | 11 | 11/46 (23.9%) |
| Susceptible | 5 | 5/20 (25.0%) |
| Control | 4 | 4/18 (22.2%) |


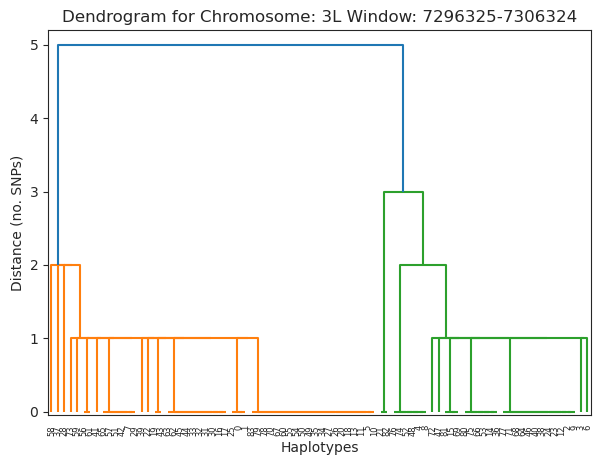


Supplementary Figure 7: Dendrogram for Chromosome 3L, Window 7296325 - 7306324

**Chromosome 3R**

Supplementary Table 11: Window 52339701 – 52349700 included two clusters of ≥20 haplotypes.

| **Window 52339701 – 52349700** **haplotype cluster (red)** | | |
| --- | --- | --- |
| Phenotype | Score | % of mosquito haplotypes |
| Resistant | 22 | 22/46 (47.8%) |
| Susceptible | 17 | 17/20 (85.0%) |
| Control | 11 | 11/18 (61.1%) |
| **Window 52339701 – 52349700** **haplotype cluster (purple)** | | |
| Phenotype | Score | % of mosquito haplotypes |
| Resistant | 14 | 14/46 (30.4%) |
| Susceptible | 2 | 2/20 (10.0%) |
| Control | 5 | 5/18 (27.8%) |

**
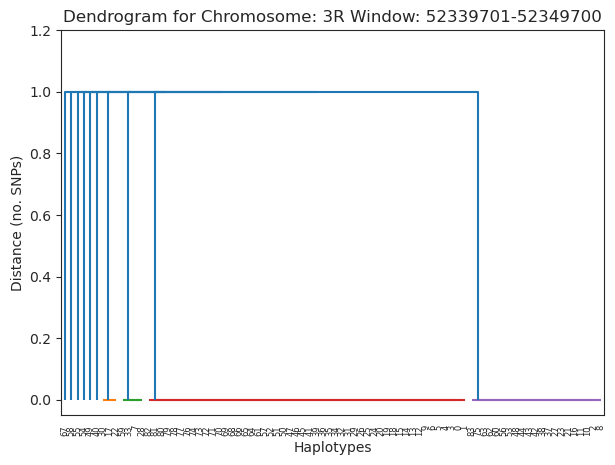
**

Supplementary Figure 8: Dendrogram for Chromosome 3R, Window 52339701-52349700

**Chromosome X**

Supplementary Table 12: Window 15832473-15842472 included one cluster of ≥20 haplotypes.

| **Window 15832473-15842472** **haplotype cluster** | | |
| --- | --- | --- |
| Phenotype | Score | % of mosquito haplotypes |
| Resistant | 14 | 14/46 (30.4%) |
| Susceptible | 4 | 4/20 (20.0%) |
| Control | 10 | 10/18 (55.6%) |


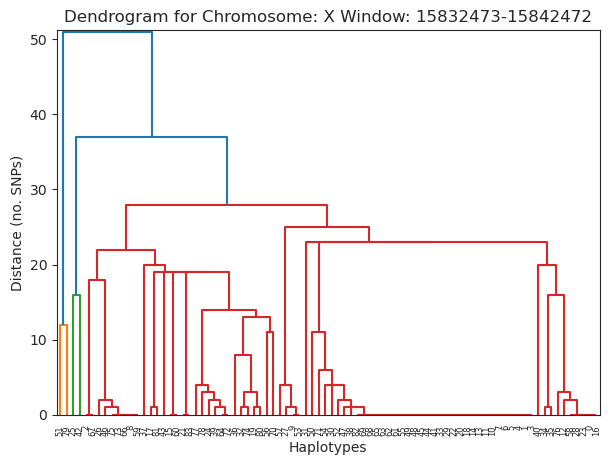


Supplementary Figure 9: Dendrogram for Chromosome X, Window 15832473 - 15842472

Supplementary Table 13: Window 15838473-15848472 had one cluster of ≥ 20 haplotypes.

| **Window 15838473-15848472 haplotype cluster** | | |
| --- | --- | --- |
| Phenotype | Score | % of mosquito haplotypes |
| Resistant | 15 | 15/46 (32.6%) |
| Susceptible | 3 | 3/20 (15.0%) |
| Control | 10 | 10/18 (55.6%) |


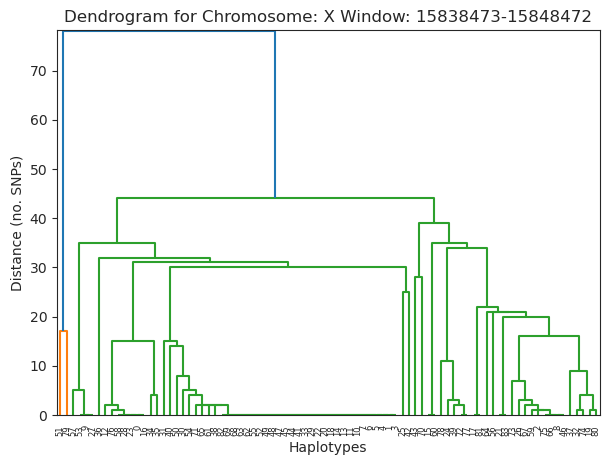


Supplementary Figure 10: Dendrogram for Chromosome X, Window 15838473 - 15848472

Supplementary Table 14: Window 15839473-15849472 had one cluster with ≥ 20 haplotypes.

| **Window 15839473-15849472 haplotype cluster** | | |
| --- | --- | --- |
| Phenotype | Score | % of mosquito haplotypes |
| Resistant | 15 | 15/46 (32.6%) |
| Susceptible | 3 | 3/20 (15.0%) |
| Control | 10 | 10/18 (55.6%) |


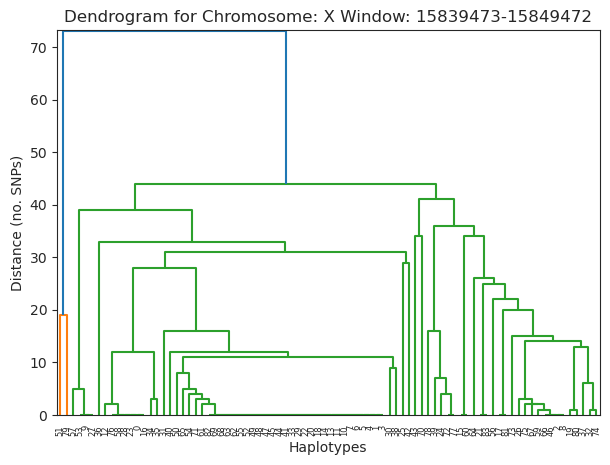


Supplementary Figure 11: Dendrogram for Chromosome X, Window 15839473-15849472

Supplementary Table 15: Window 15842473-15852472 had one cluster with ≥ 20 haplotypes

| **Window 15842473-15852472 haplotype cluster** | | |
| --- | --- | --- |
| Phenotype | Score | % of mosquito haplotypes |
| Resistant | 15 | 15/46 (32.6%) |
| Susceptible | 3 | 3/20 (15.0%) |
| Control | 10 | 10/18 (55.6%) |


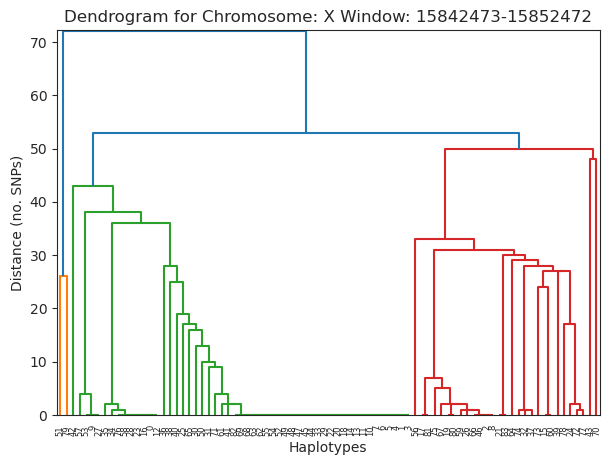


Supplementary Figure 12: Dendrogram for Chromosome X, Window 15842473-15852472

Supplementary Table 16: Window 15850473-15860472 had one cluster with ≥ 20 haplotypes.

| **Window 15850473-15860472** **haplotype cluster** | | |
| --- | --- | --- |
| Phenotype | Score | % of mosquito haplotypes |
| Resistant | 12 | 12/46 (26.1%) |
| Susceptible | 3 | 3/20 (15.0%) |
| Control | 10 | 10/18 (55.6%) |


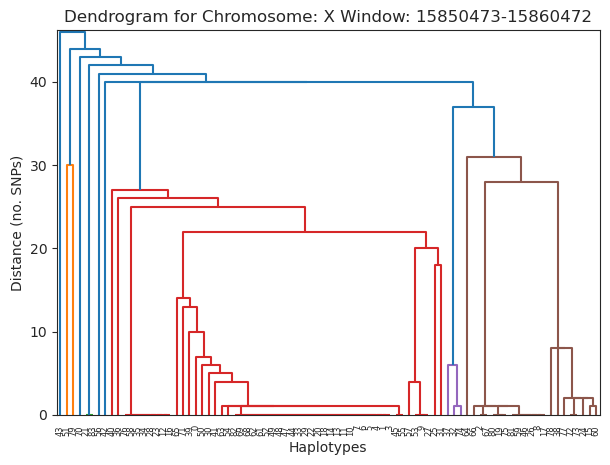


Supplementary Figure 13: Dendrogram for Chromosome X, Window 15850473 - 15860472

Supplementary Table 17: Window 15851473-15861472 had one cluster with ≥ 20 haplotypes.

| **Window 15851473-15861472 haplotype cluster** | | |
| --- | --- | --- |
| Phenotype | Score | % of mosquito haplotypes |
| Resistant | 12 | 12/46 (26.1%) |
| Susceptible | 2 | 2/20 (10.0%) |
| Control | 10 | 10/18 (55.6%) |


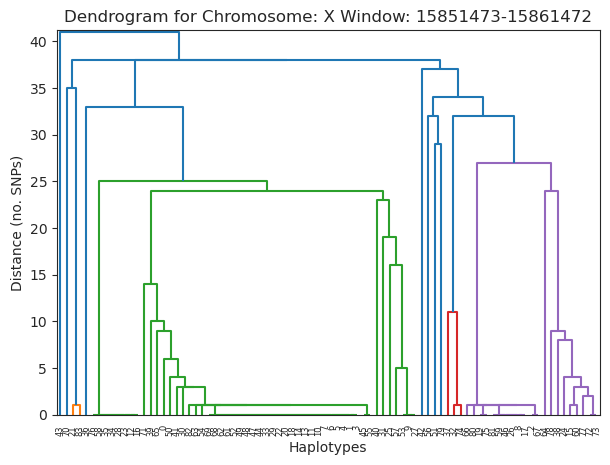


Supplementary Figure 14: Dendrogram for Chromosome X, Window 15851473 - 15861472

Supplementary Table 18: Window 15852473-15862472 had one cluster with ≥ 20 haplotypes.

| **Window 15852473-15862472 haplotype cluster** | | |
| --- | --- | --- |
| Phenotype | Score | % of mosquito haplotypes |
| Resistant | 11 | 11/46 (23.9%) |
| Susceptible | 2 | 2/20 (10.0%) |
| Control | 7 | 7/18 (38.9%) |


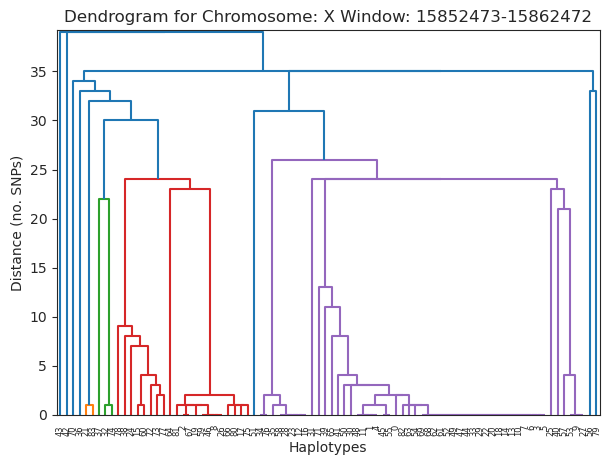


Supplementary Figure 15: Dendrogram for Chromosome X, Window 15852473-15862472

Supplementary Table 19: Window 15853473-15863472 had one cluster with ≥ 20 haplotypes.

| **Window 15853473-15863472 haplotype cluster** | | |
| --- | --- | --- |
| Phenotype | Score | % of mosquito haplotypes |
| Resistant | 10 | 10/46 (21.7%) |
| Susceptible | 2 | 2/20 (10.0%) |
| Control | 8 | 8/18 (44.4%) |


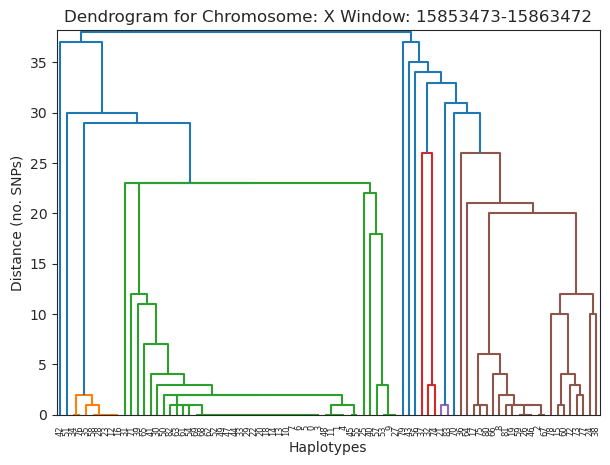


Supplementary Figure 16: Dendrogram for Chromosome X, Window 15853473-15863472

Supplementary Table 20: Window 15863473-15873472 had one cluster with ≥ 20 haplotypes.

| **Window 15863473-15873472 haplotype cluster** | | |
| --- | --- | --- |
| Phenotype | Score | % of mosquito haplotypes |
| Resistant | 21 | 21/46 (45.7%) |
| Susceptible | 4 | 4/20 (20.0%) |
| Control | 10 | 10/18 (55.6%) |


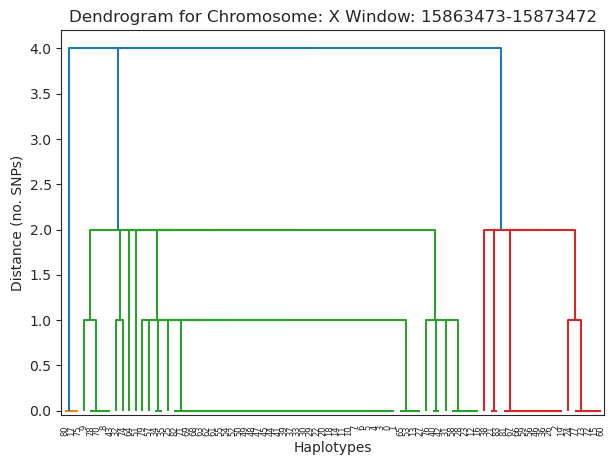


Supplementary Figure 17: Dendrogram for Chromosome X, Window 15863473 - 15873472

Supplementary Table 21: Window 16039473-16049472 had one cluster with ≥ 20 haplotypes.

| **Window 16039473-16049472 haplotype cluster** | | |
| --- | --- | --- |
| Phenotype | Score | % of mosquito haplotypes |
| Resistant | 10 | 10/46 (21.7%) |
| Susceptible | 4 | 4/20 (20.0%) |
| Control | 7 | 7/18 (38.9%) |


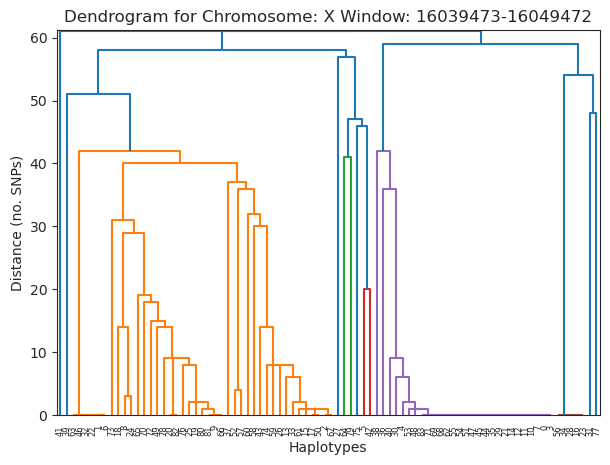


Supplementary Figure 18: Dendrogram for Chromosome X, Window 16039473 - 16049472

**Garud’s H12 Selection Scans**

**
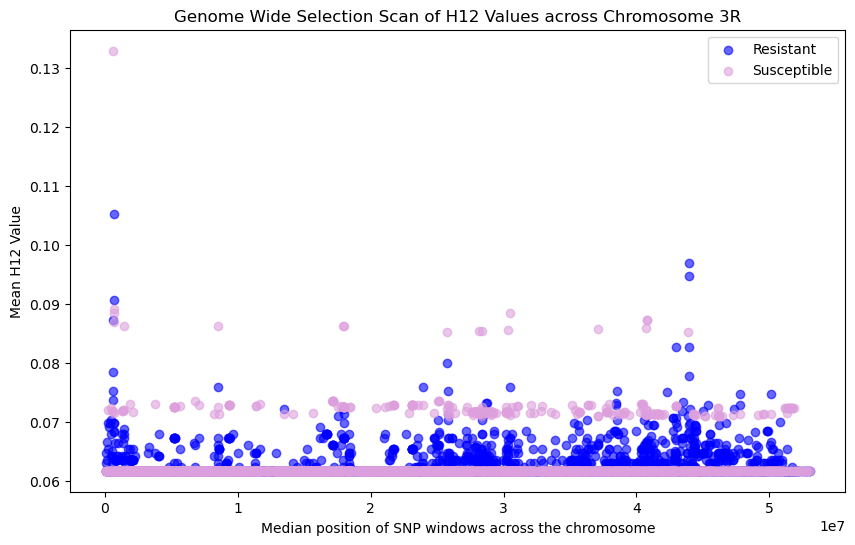
**

Supplementary Figure 19: Genome Wide Selection Scan: H12 values across chromosome 3R for resistant (blue) and susceptible (pink) mosquitoes. No clear peaks in selection were identified in resistant or susceptible mosquitoes. All H12 values were < 0.2.

**
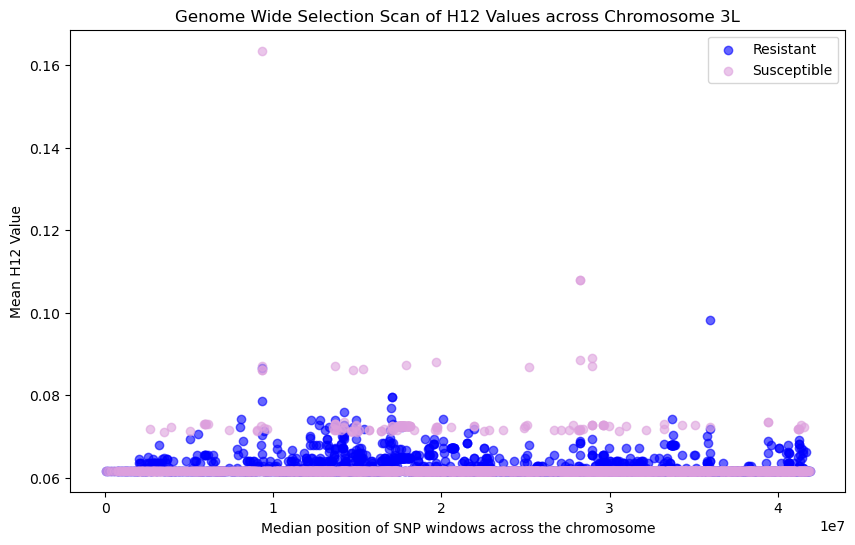
**

Supplementary Figure 20: Genome Wide Selection Scan: H12 values across chromosome 3L for resistant (blue) and susceptible (pink) mosquitoes. No clear peaks in selection were identified in resistant or susceptible mosquitoes. All H12 values were < 0.2.


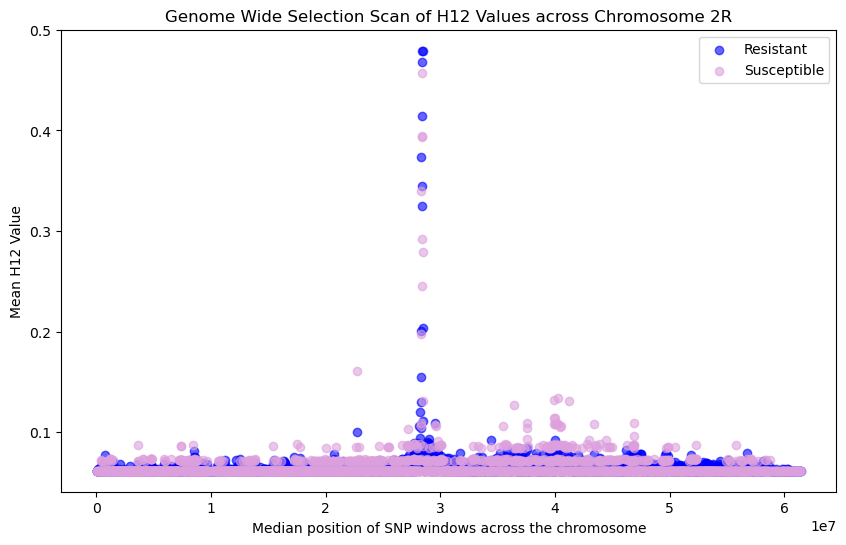


Supplementary Figure 21: Genome Wide Selection Scan: H12 values across chromosome 2R for resistant (blue) and susceptible (pink) mosquitoes. A clear peak in selection was identified. Protein coding genes overlapping this peak can be seen Supplementary Table 22.

**
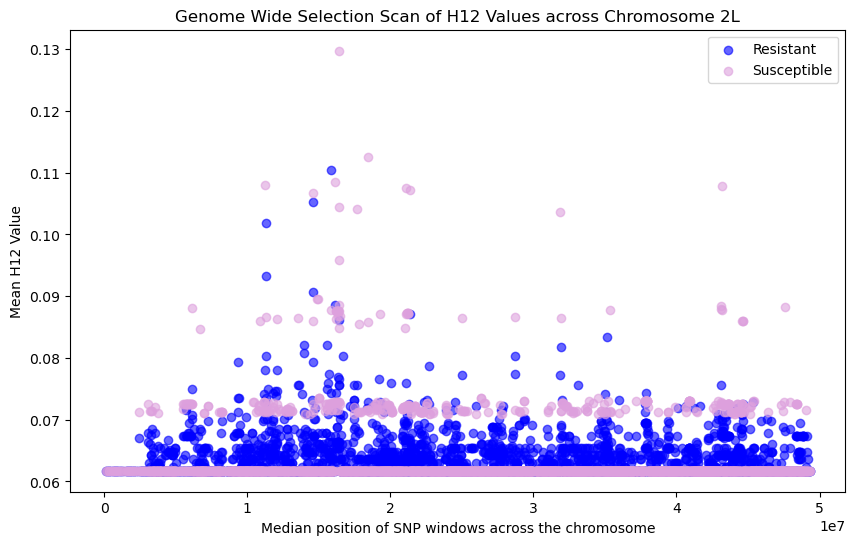
**

Supplementary Figure 22: Genome Wide Selection Scan: H12 values across chromosome 2L for resistant (blue) and susceptible (pink) mosquitoes. No clear peaks in selection were identified in resistant or susceptible mosquitoes. All H12 values were < 0.2.


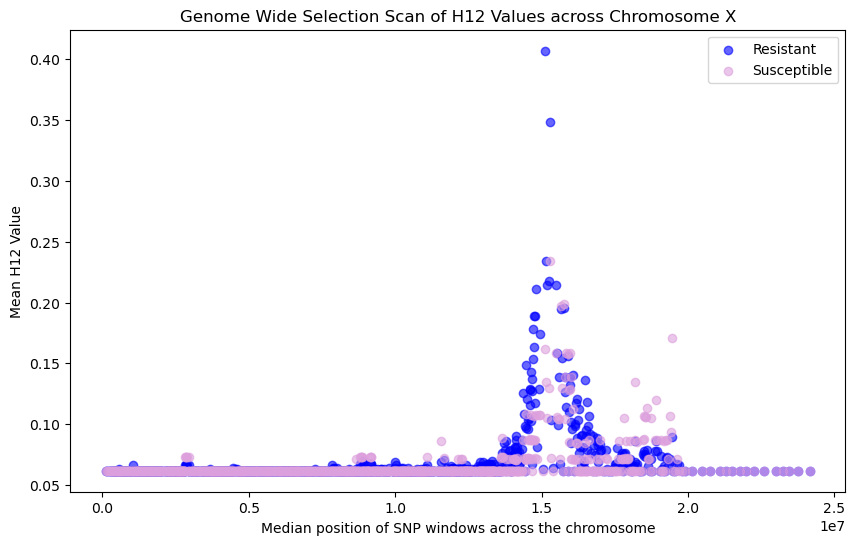


Supplementary Figure 23: Genome Wide Selection Scan: H12 values across chromosome X for resistant (blue) and susceptible (pink) mosquitoes. A clear peak in selection was identified. Protein coding genes overlapping this peak can be seen Supplementary Table 22.

Supplementary Table 22: Protein coding genes overlapping with peaks in H12 ( > 0.2).

| Chromosome | Gene ID | Description (Vector Base) |
| --- | --- | --- |
| X | AGAP000805 | BTB/POZ domain-containing protein |
|  | AGAP000806 | Angiopoietin-like 1 |
|  | AGAP000807 | Helix-loop-helix transcription factor |
|  | AGAP000808 | DNA damage-regulated autophagy modulator protein 2 |
|  | AGAP013022, AGAP000810, AGAP000814, AGAP000816, AGAP000817, AGAP013474, AGAP029892, AGAP029887, AGAP013424, AGAP013173, AGAP012997, AGAP029884, AGAP029890, AGAP029888, AGAP000821, AGAP000822, AGAP000825, AGAP000829, AGAP000834, AGAP000835, AGAP028655, AGAP013101, AGAP000848, AGAP000854, AGAP029431 | Protein coding genes, unidentified products |
|  | AGAP000809 | Proteasome 26S non-ATPase subunit 10 |
|  | AGAP000812 | Calcium binding protein |
|  | AGAP000813 | Frataxin homolog, mitochondrial |
|  | AGAP000815 | Integrin beta subunit |
|  | AGAP000818 | CYP9K1, cytochrome P450 |
|  | AGAP000819 | Nuclear receptor subfamily 2 group E member (Tailless) |
|  | AGAP000820 | CPR125, cuticular protein RR-2 family 125 |
|  | AGAP000823 | CD81 antigen |
|  | AGAP000824 | Bone morphogenetic protein 5 |
|  | AGAP000826 | cap-specific mRNA (nucleoside-2'-O-)-methyltransferase 1 |
|  | AGAP000830 | CASPS7, short caspase 7 |
|  | AGAP000831 | DnaJ homolog subfamily C member 25 |
|  | AGAP000832 | Derlin-2/3 |
|  | AGAP000833 | MIP, myoinhibitory-like peptide |
|  | AGAP013506 | UPD3A, JAK/STAT pathway cytokine unpaired 3 variant A; |
|  | AGAP000840 | Amiloride-sensitive sodium channel 2C |
|  | AGAP000841 | Ras-related protein Rab-40 |
|  | AGAP000842 | NADH dehydrogenase (ubiquinone) 1 alpha subcomplex 2C assembly factor 1 |
|  | AGAP000843 | Cardiolipin |
|  | AGAP000844 | Progestin and adipoQ receptor family member 4 |
|  | AGAP000847 | CDC-like kinase |
|  | AGAP000849 | NADH dehydrogenase (ubiquinone) 1 beta subcomplex 1 |
|  | AGAP000850 | Syx16, syntaxin 16 |
|  | AGAP000851 | Cytochrome c oxidase subunit 6 2C mitochrondrial |
|  | AGAP000852 | Small ubiquitin-related modifier |
|  | AGAP000853 | Gamma-glutamyltranspeptidase |
| 2R | AGAP002858 | Sodium/potassium-transporting ATPase subunit alpha |
|  | AGAP002859 | Solute carrier family 8 (sodium/calcium exchanger) |
|  | AGAP002862 | CYP6AA1, cytochrome P450 |
|  | AGAP013128 | CYP6AA2, cytochrome P450 |
|  | AGAP002863 | COEAE6O, carboxylesterase alpha esterase |
|  | AGAP002864 | CYP6P15P, cytochrome P450 |
|  | AGAP002865 | CYP6P3, cytochrome P450 |
|  | AGAP002866 | CYP6P5, cytochrome P450 |
|  | AGAP002867 | CYP6P4, cytochrome P450 |
|  | AGAP002868 | CYP6P1, cytochrome P450 |
|  | AGAP002869 | CYP6P2, cytochrome P450 |
|  | AGAP002870 | CYP6AD1, cytochrome P450 |
