# Supplementary material for: Phenotypic evidence of deltamethrin resistance and identification of selective sweeps in Anopheles mosquitoes on the Bijagós Archipelago, Guinea-Bissau
Source: Sci Rep. 2024 Oct 1;14:22840. doi: 10.1038/s41598-024-73996-3 (PMC11445403; doi:10.1038/s41598-024-73996-3)
Supplement: Supplementary file 3 — Supplementary Material 3 [file 41598_2024_73996_MOESM3_ESM.docx]

Allele based Odds Ratio calculations

Table 1 (from main text):

| Chromosome | Gene | Position | SNP | Resistant mosquitoes | | | | Susceptible mosquitoes | | | | Control mosquitoes | | | |
| --- | --- | --- | --- | --- | --- | --- | --- | --- | --- | --- | --- | --- | --- | --- | --- |
|  |  |  |  | Homozygous reference | Heterozygous | Homozygous alternate | No call | Homozygous reference | Heterozygous | Homozygous alternate | No call | Homozygous reference | Heterozygous | Homozygous alternate | No call |
| 2L | *vgsc* | 2416980 | T791M | 18 | 0 | 0 | 5 | 8 | 1 | 0 | 1 | 8 | 1 | 0 | 0 |
| 2L | *vgsc* | 2422652 | L995F | 15 | 7 | 0 | 0 | 7 | 3 | 0 | 0 | 6 | 3 | 0 | 0 |
| 2L | *vgsc* | 2429745 | N1570Y | 18 | 5 | 0 | 0 | 10 | 0 | 0 | 0 | 7 | 1 | 0 | 1 |
| 2L | *vgsc* | 2430424 | A1746S | 23 | 0 | 0 | 0 | 9 | 1 | 0 | 0 | 7 | 1 | 0 | 1 |
| 2L | *vgsc* | 2430881 | P1874L | 21 | 2 | 0 | 0 | 8 | 2 | 0 | 0 | 7 | 1 | 0 | 1 |
| 3R | *gste2* | 28598062 | L119V | 19 | 3 | 0 | 1 | 9 | 0 | 0 | 1 | 9 | 0 | 0 | 0 |

Odds Ratio calculations

Calculations were conducted for each of the candidate SNPs identified in our mosquito population. Within each odds ratio calculation, the number of mosquitoes that were resistant and the number that were susceptible were input into the table, separated by whether or not they carry the candidate SNP.

**T791M**

|  | Resistant phenotype | Susceptible phenotype |
| --- | --- | --- |
| Carries allele | 0 | 1 |
| Does not carry allele | 18 | 8 |

Using the Haldane-Anscombe correction.

OR = (0.5*8.5)/(1.5*18.5) = 0.153

ln(OR) = -1.876

SE(ln(OR)) = sqrt(1/0.5 + 1/1.5 + 1/18.5 + 1/8.5) = 1.685

ln(95% Confidence Interval) = -1.876 ± (1.96 x 1.685) = -1.565 ± 3.303 = -4.868 to 1.738

95% CI = e^(-4.868) to e^(1.738)

95% CI = 0.007 to 5.686

*No statistical significance.*

**L995F**

|  | Resistant phenotype | Susceptible phenotype |
| --- | --- | --- |
| Carries allele | 7 | 3 |
| Does not carry allele | 15 | 7 |

Odds Ratio = 1.086

95% Confidence Interval (0.172, 8.4)

*No statistical significance.*

**N1570Y**

|  | Resistant phenotype | Susceptible phenotype |
| --- | --- | --- |
| Carries allele | 5 | 0 |
| Does not carry allele | 18 | 10 |

Using the Haldane-Anscombe correction.

OR = (5.5*10.5)/(0.5*18.5) = 6.243

ln(OR) = 1.831

SE = sqrt(1/5.5 + 1/0.5 + 1/18.5 + 1/10.5) = 1.527

ln(95% CI) = 1.831 ± (1.96*1.527)

ln(95% CI) = 1.831 ± 2.99 = -1.159 to 4.821

95% CI = (0.314, 124.09)

*No statistical significance*

**A1746S**

|  | Resistant phenotype | Susceptible phenotype |
| --- | --- | --- |
| Carries allele | 0 | 1 |
| Does not carry allele | 23 | 9 |

Using the Haldane-Anscombe correction.

OR = (0.5*9.5)/(1.5*23.5) = 0.135

ln(OR) = -2.002

SE = sqrt(1/0.5 + 1/1 + 1/23 + 1/9) = 1.776

ln(95% CI) = -2.002 ± (1.96*1.776)

ln(95% CI) = -2.002 ± 3.481 = -5.483 to 1.479

95% CI = (0.004, 4.389)

*No statistical significance*

**P1874L**

|  | Resistant phenotype | Susceptible phenotype |
| --- | --- | --- |
| Carries allele | 2 | 2 |
| Does not carry allele | 21 | 8 |

Odds Ratio = 0.394

95% Confidence Interval (0.025, 6.287)

*No statistical significance.*

**L119V**

|  | Resistant phenotype | Susceptible phenotype |
| --- | --- | --- |
| Carries allele | 3 | 0 |
| Does not carry allele | 19 | 9 |

Using the Haldane-Anscombe correction.

OR = (3.5*9.5)/(0.5*19.5) = 3.41

ln(OR) = 1.227

SE = sqrt(1/3.5 + 1/0.5 + 1/19.5 + 1/9.5) = 1.56

ln(95% CI) = 1.227± (1.96*1.56)

ln(95% CI) = 1.227 ± 3.06 = (-1.833,4.287)

95% CI = (0.160, 72.75)

*No statistical significance*

Table 3 (from main text, includes only SNPs with an allele frequency over 5% in the sample population):

| Gene | Position | Amino acid Change | Resistant mosquitoes | | | | Susceptible mosquitoes | | | | Control mosquitoes | | | |
| --- | --- | --- | --- | --- | --- | --- | --- | --- | --- | --- | --- | --- | --- | --- |
|  |  |  | Homozygous reference | Heterozygous | Homozygous Alternate | No call | Homozygous reference | Heterozygous | Homozygous Alternate | No call | Homozygous reference | Heterozygous | Homozygous Alternate | No call |
| *gste2* | 28597956 | T154S | 5 | 13 | 4 | 1 | 2 | 5 | 1 | 2 | 3 | 5 | 1 | 0 |
| *gste2* | 28598041 | I126F | 19 | 3 | 0 | 1 | 8 | 1 | 0 | 1 | 8 | 1 | 0 | 0 |
| *gste2* | 28598505 | G26S | 20 | 2 | 0 | 1 | 8 | 1 | 0 | 1 | 9 | 0 | 0 | 0 |
| *ace1* | 3489405 | A65S | 4 | 14 | 5 | 0 | 1 | 7 | 2 | 0 | 4 | 4 | 1 | 0 |

Odds ratio calculations:

***gste2* T154S**

|  | Resistant phenotype | Susceptible phenotype |
| --- | --- | --- |
| Carries allele | 17 | 6 |
| Does not carry allele | 5 | 2 |

Odds Ratio = 1.129

95% Confidence Interval (0.0857, 9.606)

*No statistical significance.*

***gste2* I126F**

|  | Resistant phenotype | Susceptible phenotype |
| --- | --- | --- |
| Carries allele | 3 | 1 |
| Does not carry allele | 19 | 8 |

Odds Ratio = 1.254

95% Confidence Interval (0.0846,74.492)

*No statistical significance.*

***gste2* G26S**

|  | Resistant phenotype | Susceptible phenotype |
| --- | --- | --- |
| Carries allele | 2 | 1 |
| Does not carry allele | 20 | 8 |

Odds Ratio = 0.081

95% Confidence Interval (0.0369,53.029)

*No statistical significance.*

***ace1* A65S**

|  | Resistant phenotype | Susceptible phenotype |
| --- | --- | --- |
| Carries allele | 19 | 9 |
| Does not carry allele | 4 | 1 |

Odds Ratio = 0.537

95% Confidence Interval (0.009,6.556)

*No statistical significance.*
